# Dead space estimates may not be independently associated with 28-day mortality in COVID-19 ARDS

**DOI:** 10.1186/s13054-021-03570-0

**Published:** 2021-05-17

**Authors:** Luis Morales-Quinteros, Ary Serpa Neto, Antonio Artigas, Lluis Blanch, Michela Botta, David A. Kaufman, Marcus J. Schultz, Anissa M. Tsonas, Frederique Paulus, Lieuwe D. Bos, the PRoVENT-COVID Study Group, Luis Morales-Quinteros, Ary Serpa Neto, Antonio Artigas, Lluis Blanch, Michela Botta, David A. Kaufman, Marcus J. Schultz, Anissa M. Tsonas, Frederique Paulus, Lieuwe D. Bos, A. G. Algera, L. S. Boers, L. D. J. Bos, J. Pillay, D. A. Dongelmans, M. W. Hollmann, J. Horn, A. P. Vlaar, J. P. van Akkeren, A. G. Algera, C. K. Algoe, R. B. van Amstel, O. L. Baur, P. van de Berg, A. E. van den Berg, D. C. J. J. Bergmans, D. I. van den Bersselaar, F. A. Bertens, A. J. G. H. Bindels, M. M. de Boer, S. den Boer, L. S. Boers, M. Bogerd, J. S. Breel, H. de Bruin, S. de Bruin, C. L. Bruna, L. A. Buiteman–Kruizinga, O. Cremer, R. M. Determann, W. Dieperink, D. A. Dongelmans, H. S. Franke, M. S. Galek–Aldridge, M. J. de Graaff, L. A. Hagens, J. J. Haringman, S. T. van der Heide, P. L. J. van der Heiden, N. F. L. Heijnen, S. J. P. Hiel, L. L. Hoeijmakers, L. Hol, M. W. Hollmann, M. E. Hoogendoorn, J. Horn, R. van der Horst, E. L. K. Ie, D. Ivanov, N. P. Juffermans, E. Kho, E. S. de Klerk, A. W. M. M. Koopman-van Gemert, M. Koopmans, S. Kucukcelebi, M. A. Kuiper, D. W. de Lange, N. van Mourik, S. G. Nijbroek, M. Onrust, E. A. N. Oostdijk, C. J. Pennartz, J. Pillay, L. Pisani, I. M. Purmer, T. C. D. Rettig, J. P. Roozeman, M. T. U. Schuijt, M. E. Sleeswijk, M. R. Smit, P. E. Spronk, W. Stilma, A. C. Strang, P. R. Tuinman, C. M. A. Valk, F. L. Veen-Schra, L. I. Veldhuis, P. van Velzen, W. H. van der Ven, A. P. J. Vlaar, P. van Vliet, P. H. J. van der Voort, L. van Welie, H. J. F. T. Wesselink, H. H. van der Wier-Lubbers, B. van Wijk, T. Winters, W. Y. Wong, A. R. H. van Zanten

**Affiliations:** 1 Intensive Care Unit, Hospital Universitari General de Catalunya, Grupo Quironsalud, Carrer Pedro i Pons, 1, 08195 Sant Cugat del Vallès, Barcelona, Spain; 2 Universidad Autonoma de Barcelona, Barcelona, Spain; 3 Institut D’Investigació, Innovació Parc Taulí I3PT, Sabadell, Spain; 4 Department of Intensive Care & Laboratory of Experimental Intensive Care and Anaesthesiology (L·E·I·C·A), Amsterdam UMC Location AMC, Amsterdam, The Netherlands; 5 Department of Critical Care Medicine, Hospital Israelita Albert Einstein, São Paulo, Brazil; 6 Australian and New Zealand Intensive Care Research Centre (ANZIC-RC), Monash University, Melbourne, Australia; 7 Data Analytics Research and Evaluation (DARE) Centre, Austin Hospital and University of Melbourne, Melbourne, Australia; 8 Critical Care Center, Corporacion Sanitaria Universitaria Parc Taulí, Sabadell, Spain; 9 CIBER Enfermedades Respiratorias (ISCiii), Madrid, Spain; 10 Division of Pulmonary, Critical Care, and Sleep Medicine, NYU School of Medicine, New York, NY USA; 11 Nuffield Department of Medicine, Oxford University, Oxford, UK; 12 Mahidol-Oxford Tropical Medicine Research Unit (MORU), Mahidol University, Bangkok, Thailand

**Keywords:** Acute respiratory distress syndrome, ARDS, Respiratory dead space, Dead space, Ventilatory ratio, COVID-19, Mortality, Prognostication

## Abstract

**Background:**

Estimates for dead space ventilation have been shown to be independently associated with an increased risk of mortality in the acute respiratory distress syndrome and small case series of COVID-19-related ARDS.

**Methods:**

Secondary analysis from the PRoVENT-COVID study. The PRoVENT-COVID is a national, multicenter, retrospective observational study done at 22 intensive care units in the Netherlands. Consecutive patients aged at least 18 years were eligible for participation if they had received invasive ventilation for COVID-19 at a participating ICU during the first month of the national outbreak in the Netherlands. The aim was to quantify the dynamics and determine the prognostic value of surrogate markers of wasted ventilation in patients with COVID-19-related ARDS.

**Results:**

A total of 927 consecutive patients admitted with COVID-19-related ARDS were included in this study. Estimations of wasted ventilation such as the estimated dead space fraction (by Harris–Benedict and direct method) and ventilatory ratio were significantly higher in non-survivors than survivors at baseline and during the following days of mechanical ventilation (*p* < 0.001). The end-tidal-to-arterial PCO_2_ ratio was lower in non-survivors than in survivors (*p* < 0.001). As ARDS severity increased, mortality increased with successive tertiles of dead space fraction by Harris–Benedict and by direct estimation, and with an increase in the VR. The same trend was observed with decreased levels in the tertiles for the end-tidal-to-arterial PCO_2_ ratio. After adjustment for a base risk model that included chronic comorbidities and ventilation- and oxygenation-parameters, none of the dead space estimates measured at the start of ventilation or the following days were significantly associated with 28-day mortality.

**Conclusions:**

There is significant impairment of ventilation in the early course of COVID-19-related ARDS but quantification of this impairment does not add prognostic information when added to a baseline risk model.

*Trial registration*: ISRCTN04346342. Registered 15 April 2020. Retrospectively registered.

**Supplementary Information:**

The online version contains supplementary material available at 10.1186/s13054-021-03570-0.

## Background

Since the outbreak of coronavirus disease 2019 (COVID-19) in the City of Wuhan, Hubei Province, China, caused by the transmission of the novel coronavirus SARS-CoV-2, millions of individuals have been infected and more than one million have died. Severe disease requiring admission to intensive care unit (ICU) occurs in approximately 5% of infections [1], and the most common reason for admission is respiratory failure requiring high-level support. Among these patients, two-thirds meet the criteria for the acute respiratory distress syndrome (ARDS) [2].

Patients with COVID-19 pneumonia meeting criteria for ARDS usually present with a high respiratory drive and minute ventilation, potentially due to hypercapnia and an increased dead space fraction (*V*_D_/*V*_T_) [3]. In patients with ARDS, an elevated *V*_D_/*V*_T_ is a predictor of death and is one of the few lung-specific physiological variables independently associated with mortality [4, 5]. Methods for estimating *V*_D_/*V*_T_ do not require quantitative assessment of exhaled carbon dioxide, are less complicated to perform and easier to calculate at the bedside compared with calculations made by volumetric capnography [6]. In recent years, the ventilatory ratio (VR) was proposed as an easily acquired bedside index of impaired ventilation that can be computed using routinely measured respiratory variables [7]. In patients with ARDS, the VR correlates well with *V*_D_/*V*_T_ [7] and may function as a surrogate marker for impaired ventilation [8].

At least two independent groups have described series of patients with COVID-19-related ARDS who may have inefficient CO_2_ removal due to increased physiologic dead space [3, 9]. However, few studies have assessed the impact for dead space ventilation mortality in a large cohort of COVID-19 patients undergoing invasive ventilation [10]. Therefore, we aimed to assess the association between markers of impaired ventilation, such as estimated *V*_D_/*V*_T_ and VR with 28-day mortality in patients undergoing invasive ventilation because of COVID-19 ARDS. We hypothesized that these markers of impaired ventilation are independently associated with 28-day mortality.

## Methods

### Study design and oversight

PRoVENT-COVID is an investigator-initiated, multicenter, observational cohort study undertaken at 22 ICUs in the Netherlands. The study protocol including the statistical analysis plan is available [11]. The approved protocol is available in Additional File 1. A statistical analysis plan for the current analysis was written before assessing the database and is available online [12]. Study sites were recruited through direct contact by members of the steering committee of PRoVENT-COVID. The institutional review boards of the participating centers approved the study protocol, and need for patient informed consent was waived. Study coordinators contacted the local doctors, trained data collectors to assist the local doctors and monitored the study according to the International Conference on Harmonization Good Clinical Practice–guidelines. Integrity and timely completion of data collection was ensured by the study coordinators.

### Patients

Consecutive patients ≥ 18 years of age were eligible for participation in PRoVENT-COVID if they were admitted to one of the participating ICUs and had received invasive ventilation for COVID-19 ARDS. COVID-19 infection was defined by a confirmed reverse transcriptase-polymerase chain reaction (RT-PCR) [13].

PRoVENT-COVID had no exclusion criteria, but for the current analysis, we excluded patients transferred from a non-participating hospital who had been receiving invasive ventilation for more than 2 calendar days, patients without complete data to calculate the V_D_/V_T_ or VR on the first day of ventilation, and patients with no data about 28-day mortality.

### Data collection

Demographics and data regarding premorbid diseases and medication were collected at baseline. Ventilator settings and parameters were collected after one hour of invasive ventilation and every 8 h thereafter, for the first 4 calendar days. In the present study, the first day of ventilation is called ‘at start of ventilation.’

### Data definition and exposure

The primary exposure of interest was the V_D_/V_T_ calculated using the Harris–Benedict formula as described in Eq. (1) [14]:

1 $$\frac{{V_{{\text{D}}} }}{{V_{{\text{T}}} }} = 1 - \frac{{\left( {0.863* \dot{V}{\text{CO}}_{2} } \right)}}{{\left( {{\text{RR}}* V_{{\text{T}}} * {\text{PaCO}}_{2} } \right)}}$$

RR is the respiratory rate in breaths per minute, V_T_ the tidal volume in liters, PaCO_2_ the partial pressure of carbon dioxide in mmHg, and VCO_2_ the CO_2_ production in mL/min estimated using Eq. (2):

2 $$\dot{V}{\text{CO}}_{2} = \frac{{{\text{REE}}_{{{\text{HB}}}} }}{{\left( {\frac{5.616}{{{\text{RQ}}}} + 1.584} \right)}}$$

RQ is the respiratory quotient, assumed to be 0.8, and REE_HB_ is the rest energy expenditure calculated by the unadjusted Harris–Benedict estimate using Eq. (3) [14]:

3 $$\begin{aligned} {\text{Males}}:{\text{REE}}_{{{\text{HB}}}} & = 66.473 + \left( {13.752*{\text{weight}}} \right) + \left( {5.003*{\text{height}}} \right) - \left( {6.755*{\text{age}}} \right) \\ {\text{Females}}:{\text{REE}}_{{{\text{HB}}}} & = 655.096 + \left( {9.563*{\text{weight}}} \right) + \left( {1.850*{\text{height}}} \right) - \left( {4.676*{\text{age}}} \right) \\ \end{aligned}$$

Weight is the actual body weight in kilograms, height is in centimeters and age in years.

In addition, two additional estimations of V_D_/V_T_ were done considering a direct estimation [6] and the end-tidal-to-arterial PCO_2_ ratio [15], and the formulas are described in the Additional File 1.

The secondary exposure of interest is the VR, calculated using Eq. (4) [16]:

4 $${\text{VR}} = \frac{{\dot{V}_{{E {\text{measured}}}} * {\text{PaCO}}_{{2 {\text{measured}}}} }}{{\dot{V}_{{E {\text{predicted}}}} * {\text{PaCO}}_{{2 {\text{predicted}}}} }}$$

VR is the ventilatory ratio, *V*_E measured_ is the measured minute ventilation in mL/min, PaCO_2 measured_ is the measured PaCO_2_ in mmHg, *V*_E predicted_ is the predicted minute ventilation in mL/min (calculated as 100 * predicted body weight) [16], and PaCO_2 predicted_ is the predicted PaCO_2_ determined as 37.5 mmHg.

A post-hoc analysis was performed using Corrected Minute Ventilation as additional parameter of wasted ventilation. This parameter is calculated using the following formula:

5 $$\dot{V}_{{{\text{E}}\,{\text{corr}}}} = \frac{{\dot{V}_{E } * {\text{PaCO}}_{2 } }}{{40\, {\text{mmHg}}}}$$

where 40 mm Hg is the ideal value of PaCO_2_ [17]. This is reported in the Additional File 1. Additionally, the delta values between days were calculated and used as additional parameters and reported in the Additional File 1.

All variables were calculated three times per day, and the values were aggregated as the mean in the respective day. Primary analyses focused on the values obtained on the day on which ventilation was started.

### Outcomes

The outcome assessed in this study was death at 28 days, defined as the mortality 28 days after the start of ventilation. Other clinical outcomes are reported only to describe the cohort but were not used to test their association with the exposures described above.

### Statistical analysis

The amount of missing data was low for most of the variables (Table S1 in Additional File 1). Continuous variables are presented as median (quartile 25%–quartile 75%) and categorical variables as counts and percentages. Descriptive data are presented according to the 28-day status (non-survivors vs. survivors), and the two groups were compared using Wilcoxon rank-sum test for continuous variables, and Fisher exact tests for categorical variables.

Trends in markers of impaired ventilation were presented in boxplots between survivors and non-survivors over the first 4 calendar days. The direction of effect over time of the variables was assessed with mixed–effect linear models with center and patients treated as random effect to account for clustering and repeated measurements, and with 28-day vital status (alive/dead), time (as a continuous variable) and an interaction of 28-day vital status and time as fixed effect. Overall *P* values from this analysis represent the overall difference among groups over time, and *P* values from interaction represent a statistical assessment of whether the trend over time differed among the groups. All daily measurements of variables (three times a day) were aggregated as the mean per day. In addition, to compare variables at each day, the day variable was entered as a categorical variable in the model described above, and the *P* value for the daily difference was extracted using pairwise comparisons after Bonferroni correction.

We examined the risk of death for each tertile of the lung-specific physiological variables used to evaluate whether the predictive ability of each variable varied by level. In addition, a simple stratification creating two groups according to the median of the variables was also assessed. The comparison of the two groups was presented in Kaplan–Meier curves and compared using Log-rank tests.

Univariable mixed-effect generalized linear models considering a binomial distribution and with center as random effect were used to estimate the unadjusted effect of each variable on 28-day mortality. A multivariable mixed-effect generalized linear model considering a binomial distribution and with center as random effect were used to test the association of each of the exposures described above with 28-day mortality. A list of candidate confounders was determined a priori, and based on clinical relevance rather than statistical significance. The following baseline variables (measured at baseline or within 1 h after intubation or ICU admission with ventilation) were considered in the models: age, gender, body mass index, PaO_2_/FiO_2_ ratio, plasma creatinine, hypertension, diabetes, use of angiotensin converting enzyme inhibitors, use of angiotensin II receptor blockers, use of a vasopressor or an inotrope drug, fluid balance, pH, mean arterial pressure, heart rate, respiratory system compliance and PEEP. Multicollinearity was assessed through the analysis of the variance inflation factors, and the final model was assessed for discrimination using c-statistics, and for calibration using the Brier-Score.

In addition to the odds ratio (OR) and its 95% confidence interval, the predictive accuracy of the lung-specific physiological variables was measured by the area under the receiver operating characteristics curve (AUC-ROC). Also, to estimate whether these variables improved predictive accuracy on top of that of the base model described above, the net reclassification improvement (NRI) and the integrated discrimination index (IDI) were assessed.

For the primary analysis, covariates with less than 3% of missing values were imputed by the median value of the overall cohort. Since respiratory compliance was missing in 8.2% of the patients (Table S1 in Additional File 1), an additional sensitivity analysis considering multiple imputation for all missing variables was conducted (described in details in Additional File 1).

All continuous variables were entered after standardization to improve convergence of the models, and the odds ratio (OR) represents the increase in one standard deviation of the variable. All analyses were conducted in R v.4.0.2 (R Foundation, Vienna, Austria) [18], and significance level was set at 0.05.

## Results

### Study population

From March 1, 2020, through June 1, 2020, 31 ICUs were invited for participation in PRoVENT-COVID, and 22 of them met inclusion criteria. A total of 1340 individuals were screened. A total of 218 were not enrolled; 62 did not have COVID-19-related ARDS, 150 never received invasive ventilation, and 6 were excluded for other reasons (Additional File 1: Figure S1). Of the enrolled 927 patients, 259 (28.7%) were non-survivors and 661 (71.3%) were survivors at day 28. Demographics characteristics are presented in Table 1. Non-survivors were older, more often male, had a greater severity of ARDS and higher creatinine level at admission, more frequent presented co-existing disorders like hypertension, diabetes and chronic obstructive pulmonary disease, and more often were using systemic steroids, metformin, beta-blockers and statins at home. Ventilatory variables in the first day of ventilation and general clinical outcomes are shown in Table 2, Table S2 and Table S3 (Additional File 1). Non-survivors received higher FiO_2_, had higher levels of lactate and creatinine and lower levels of pH and end-tidal-to-arterial PCO_2_. There were no differences in the radiographic findings between survivors and non-survivors (Table S15).

**Table 1:** Baseline characteristics of the patients according to 28-day mortality

	All patients (*n* = 927)	Non-survivors (*n* = 266)	Survivors (*n* = 661)	*p* value
Age, years	65.0 (57.0–72.0)	70.0 (64.0–75.0)	63.0 (55.0–70.0)	< 0.001
Male gender—no (%)	668 (72.1)	206 (77.4)	462 (69.9)	0.023
Body mass index, kg/m^2^	27.8 (25.3–30.8)	27.7 (25.2–29.8)	27.8 (25.4–30.9)	0.227
Severity of ARDS—no (%)				0.020
Mild	186/921 (20.2)	45 (17.0)	141 (21.5)	
Moderate	638/921 (69.3)	180 (68.2)	458 (69.7)	
Severe	97/921 (10.5)	39 (14.8)	58 (8.8)	
Co-existing disorders—no (%)				
Hypertension	317 (34.2)	112 (42.1)	205 (31.0)	0.002
Heart failure	39 (4.2)	16 (6.0)	23 (3.5)	0.102
Diabetes	207 (22.3)	78 (29.3)	129 (19.5)	0.002
Chronic kidney disease	41 (4.4)	16 (6.0)	25 (3.8)	0.157
Baseline creatinine, µmol/L*	76.0 (62.0–97.0)	84.0 (65.0–112.0)	74.0 (61.0–92.0)	< 0.001
Liver cirrhosis	3 (0.3)	0 (0.0)	3 (0.5)	0.562
Chronic obstructive pulmonary disease	76 (8.2)	32 (12.0)	44 (6.7)	0.011
Active hematological neoplasia	15 (1.6)	6 (2.3)	9 (1.4)	0.388
Active solid neoplasia	23 (2.5)	9 (3.4)	14 (2.1)	0.253
Neuromuscular disease	8 (0.9)	3 (1.1)	5 (0.8)	0.696
Immunosuppression	24 (2.6)	7 (2.6)	17 (2.6)	0.999
Previous medication—no (%)				
Systemic steroids	35 (3.8)	17 (6.4)	18 (2.7)	0.012
Inhalation steroids	108 (11.7)	40 (15.0)	68 (10.3)	0.054
Angiotensin converting enzyme inhibitor	160 (17.3)	55 (20.7)	105 (15.9)	0.084
Angiotensin II receptor blocker	101 (10.9)	35 (13.2)	66 (10.0)	0.164
Vital signs				
Heart rate, bpm	84.3 (73.7–97.0)	85.0 (74.5–100.3)	84.0 (73.5–96.0)	0.092
Mean arterial pressure, mmHg	80.3 (73.7–88.0)	80.0 (73.0–86.0)	80.5 (74.8–88.3)	0.061
Organ support—no (%)				
Continuous sedation	892 (96.4)	252 (95.1)	640 (97.0)	0.173
Inotropic or vasopressor	733 (79.2)	216 (81.5)	517 (78.3)	0.324
Vasopressor	732 (79.1)	216 (81.5)	516 (78.2)	0.283
Inotropic	41 (4.4)	17 (6.4)	24 (3.6)	0.077
Fluid balance, mL	631.5 (50.0–1428.2)	753.6 (100.0–1482.0)	593.0 (34.0–1390.0)	0.143
Urine output, mL	702.5 (370.0–1143.8)	570.0 (280.0–1075.0)	725.0 (415.0–1165.0)	0.001

Data are median (quartile 25%—quartile 75%) or No (%). Percentages may not total 100 because of rounding

* Most recent measurement in 24 h before intubation, or at ICU admission under invasive ventilation

**Table 2:** Respiratory variables at start of ventilation

	All Patients (*n* = 927)	Non-Survivors (*n* = 266)	Survivors (*n* = 661)	*p* value
Tidal volume, mL/kg PBW	6.4 (5.9–7.1)	6.5 (5.9–7.2)	6.4 (5.9–7.1)	0.175
PEEP (cmH_2_O)	12.7 (11.0–14.5)	13.0 (11.2–15.0)	12.7 (10.7–14.3)	0.091
Driving pressure (cmH_2_O)	14.0 (12.0–16.0)	13.7 (12.0–16.3)	14.0 (12.0–16.0)	0.746
PaO_2_/FiO_2_	130.9 (99.9–175.5)	137.2 (98.1–180.0)	128.8 (100.2–171.5)	0.262
EtCO_2_	36.5 (32.6–41.6)	34.7 (31.7–40.2)	37.0 (33.2–42.0)	0.001
Mechanical power (J/min)	18.4 (15.2–22.3)	18.9 (15.9–22.8)	18.3 (15.1–22.0)	0.100
Compliance (mL/cmH_2_O)	33.2 (26.7–40.3)	33.8 (26.7–40.7)	32.9 (26.7–40.1)	0.899

Data are median (quartile 25%—quartile 75%). FiO_2_: inspired fraction of oxygen; PEEP: positive end-expiratory pressure

### Markers of impaired ventilation

**Fig. 1:**
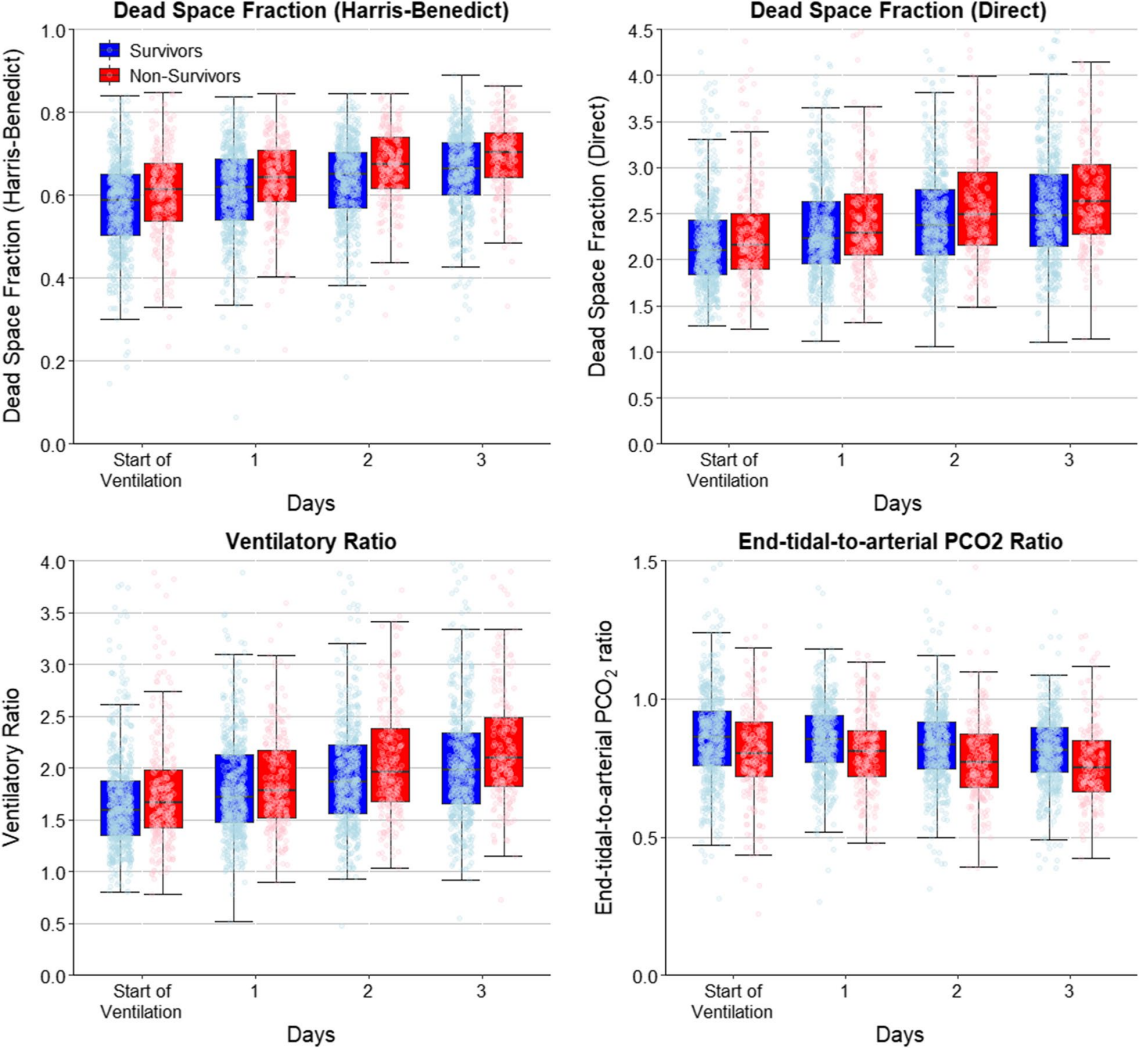
Lung-specific physiological variables over the first four days of ventilation. Jitter boxplot of lung-specific physiological variables over the first four days of ventilation

The dynamic change of markers of impaired ventilation over the first four days of ventilation as shown in Table 3 and Fig. 1. *V*_D_/*V*_T_ calculated using the Harris–Benedict formula was higher in non-survivors and increased more during the first four days compared to survivors. A similar trend was found with the direct *V*_D_/*V*_T_ calculation. While VR was also higher in non-survivors, especially after day 2, the end-tidal-to-arterial PCO_2_ ratio was lower.

**Table 3:** Lung-specific physiological variables in the first four days of ventilation according to 28-day mortality

	All Patients (*n* = 927)	Non-Survivors (*n* = 266)	Survivors (*n* = 661)	*p* value*
Dead space fraction by HB				
At start of ventilation	0.58 ± 0.11	0.60 ± 0.11	0.58 ± 0.11	< 0.001
Day 01	0.62 ± 0.10	0.64 ± 0.09	0.61 ± 0.11	< 0.001
Day 02	0.64 ± 0.10	0.67 ± 0.09	0.63 ± 0.10	< 0.001
Day 03	0.67 ± 0.09	0.69 ± 0.08	0.65 ± 0.10	< 0.001
*p* value (interaction survival × day)		0.005		
Dead space fraction direct				
At start of ventilation	2.22 ± 0.55	2.28 ± 0.61	2.19 ± 0.52	0.022
Day 01	2.35 ± 0.54	2.40 ± 0.55	2.33 ± 0.54	0.036
Day 02	2.48 ± 0.58	2.60 ± 0.63	2.44 ± 0.54	< 0.001
Day 03	2.62 ± 0.65	2.77 ± 0.71	2.56 ± 0.61	< 0.001
*p* value (interaction survival × day)		< 0.001		
Ventilatory ratio				
At start of ventilation	1.72 ± 0.60	1.77 ± 0.56	1.70 ± 0.62	0.114
Day 01	1.85 ± 0.64	1.88 ± 0.53	1.84 ± 0.68	0.142
Day 02	1.99 ± 0.66	2.09 ± 0.60	1.95 ± 0.68	< 0.001
Day 03	2.12 ± 0.70	2.26 ± 0.68	2.06 ± 0.70	< 0.001
*p* value (interaction survival × day)		< 0.001		
end-tidal-to-arterial PCO_2_ ratio				
At start of ventilation	0.85 ± 0.18	0.81 ± 0.18	0.87 ± 0.18	< 0.001
Day 01	0.84 ± 0.14	0.81 ± 0.14	0.85 ± 0.13	< 0.001
Day 02	0.82 ± 0.14	0.78 ± 0.15	0.83 ± 0.14	< 0.001
Day 03	0.80 ± 0.15	0.77 ± 0.15	0.81 ± 0.14	< 0.001
*p* value (interaction survival × day)		0.455		

Data are median (quartile 25%—quartile 75%)

HB: Harris–Benedict

*Calculated using pairwise contrasts in a mixed-effect generalized linear model considering a Gaussian distribution and with day, group and an interaction day × group as fixed effect, and with patients and center as random effect. A binomial distribution was used for binary variables and a Gaussian distribution for continuous

**Fig. 2:**
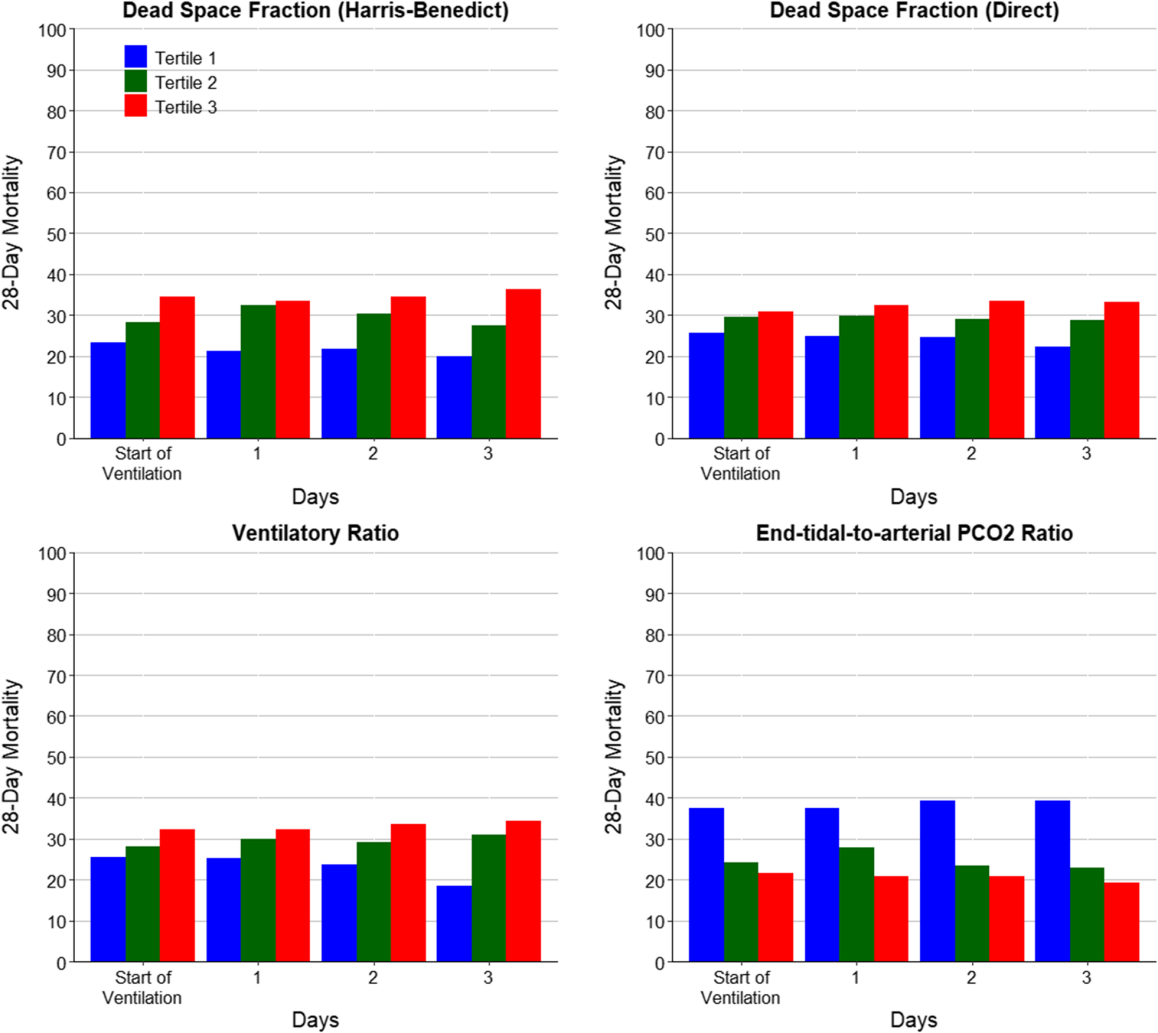
28-Day mortality according to tertiles of lung-specific physiological variables over the first four days of ventilation. **a** Tertiles are < 0.54, 0.54–0.64 and > 0.64 for start of ventilation, < 0.58, 0.58–0.67, > 0.67 for day 1, < 0.62, 0.62–0.69, > 0.69 for day 2, and < 0.64, 0.64–0.71, > 0.71 for day 3; **b** tertiles are < 1.94, 1.94–2.32 and > 2.32 for start of ventilation, < 2.09, 2.09–2.47, > 2.47 for day 1, < 2.19, 2.19–2.65, > 2.65 for day 2, and < 2.31, 2.31–2.80, > 2.80 for day 3; **c** tertiles are < 1.45, 1.45–1.80 and > 1.80 for start of ventilation, < 1.57, 1.57–1.98, > 1.98 for day 1, < 1.71, 1.71–2.13, > 2.13 for day 2, and < 1.80, 1.20—2.26, > 2.26 for day 3; and **d** tertiles are < 0.77, 0.77—0.91 and > 0.91 for start of ventilation, < 0.79, 0.79–0.90, > 0.90 for day 1, < 0.76, 0.76–0.87, > 0.87 for day 2, and < 0.74, 0.74—0.85, > 0.85 for day 3

Mortality by tertiles of each variable is reported in Fig. 2. Tertiles were calculated separately for each variable and each day, to account for potential differences in scaling and measurements. Mortality increased with successive tertiles of dead space fraction by Harris–Benedict and by direct estimation, and of ventilatory ratio, and decreased with successive tertiles of end-tidal-to-arterial PCO_2_ ratio.

**Fig. 3:**
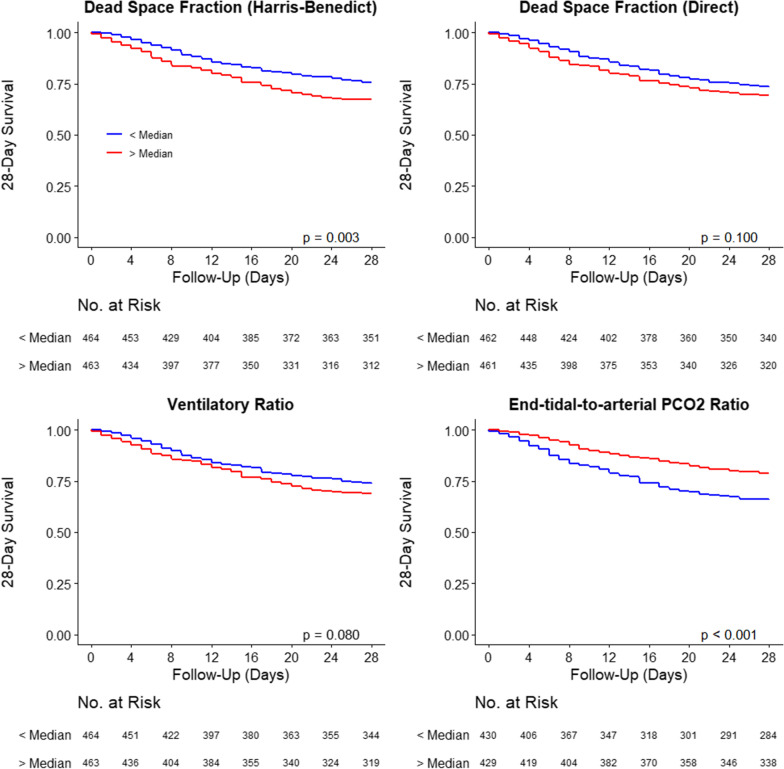
28-Day survival according to lung-specific physiological variables measured at the start of ventilation. Groups were created according to the median of the variables at start of ventilation; *p* values from Log-rank tests

Mortality over the first 28 days was higher in patients in the high group of dead space fraction by the Harris–Benedict estimation (16.4% vs. 12.3%; *p* = 0.003), but not different in the groups considering the dead space fraction by direct estimation (15.4% vs. 13.3%; *p* = 0.100), and the ventilatory ratio (15.5% vs. 13.2%; *p* = 0.080) (Fig. 3). When assessing the end-tidal-to-arterial PCO_2_ ratio, 28-day mortality was lower in the highest tertile group (10.7% vs. 17.1%; *p* < 0.001).

### Predictive accuracy of markers of impaired ventilation

The unadjusted impact of each marker of impaired ventilation is shown in Additional File 1: Table S4. Estimated dead space fraction (by HB and direct method) and end-tidal-to-arterial PCO_2_ ratio were associated with 28-day mortality at the start of mechanical ventilation. Twenty-four hours after this, dead space fraction by Harris–Benedict and end-tidal-to-arterial PCO_2_ ratio were associated with 28-day mortality. The final multivariable base risk model is shown in Table S5 and in the Additional File 1. No problems were found due to multicollinearity or linearity assumption (Additional File 1: Tables S6 and S7).

**Fig. 4:**
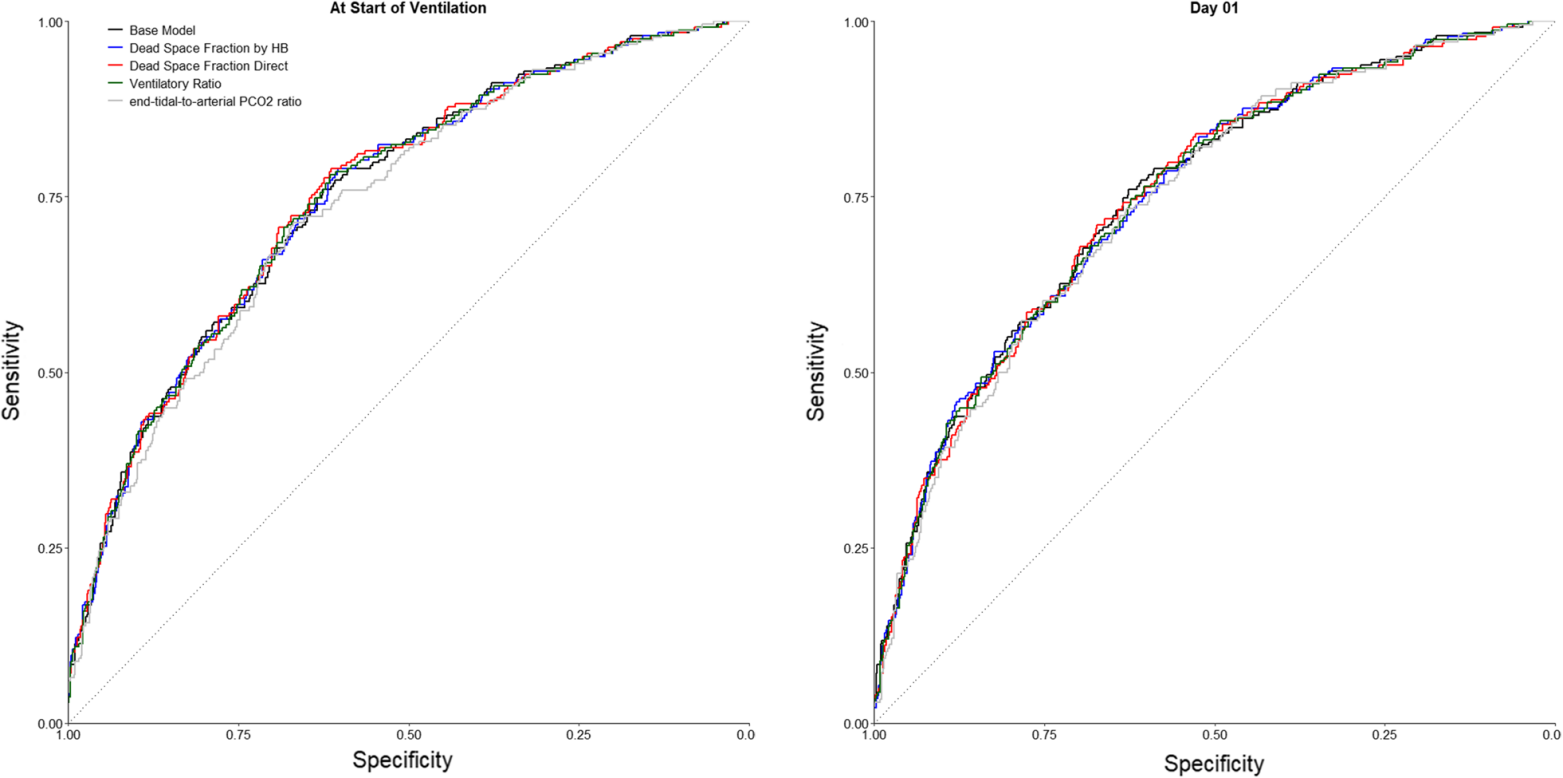
Receiver operating characteristics curve of the base model and with the inclusion of lung-specific physiological variables

After adjustment for the base risk model, none of the markers of impaired ventilation measured at the start of ventilation or the following day was significantly associated with 28-day mortality (Table 4). The inclusion of these variables did not improve the AUC-ROC compared to the base model (Fig. 4). The addition of dead space fraction by direct estimation at start of ventilation and of end-tidal-to-arterial PCO_2_ ratio at start of ventilation or day 1 slightly improved the predictive accuracy of the base model in terms of IDI (Table 4).

**Table 4:** Predictive accuracy of lung-specific physiological variables

	Odds RATIO* (95% CI)	*p* value	AUC (95% CI)	Brier Score	NRI (95% CI)	*p* value	IDI (95% CI)	*p* value
Base model	–	–	0.751 (0.715 to 0.788)	0.167	–	–	–	
At start of ventilation								
+ Dead space fraction by HB	0.91 (0.74 to 1.11)	0.356	0.751 (0.715 to 0.788)	0.167	0.08 (− 0.07 to 0.23)	0.280	0.001 (− 0.000 to 0.003)	0.165
+ Dead space fraction direct	0.83 (0.67 to 1.02)	0.082	0.754 (0.718 to 0.790)	0.167	0.13 (− 0.02 to 0.28)	0.081	0.005 (− 0.000 to 0.009)	0.025
+ Ventilatory ratio	0.90 (0.71 to 1.14)	0.394	0.752 (0.716 to 0.788)	0.167	0.07 (− 0.08 to 0.22)	0.350	0.001 (− 0.000 to 0.004)	0.067
+ end-tidal-to-arterial PCO_2_ ratio	0.93 (0.76 to 1.15)	0.521	0.739 (0.701 to 0.778)	0.168	− 0.13 (− 0.28 to 0.03)	0.106	− 0.009 (− 0.015 to − 0.003)	0.001
Day 01								
+ Dead space fraction by HB	1.05 (0.86 to 1.29)	0.619	0.749 (0.712 to 0.786)	0.170	− 0.07 (− 0.22 to 0.08)	0.362	− 0.000 (− 0.003 to 0.004)	0.864
+ Dead space fraction direct	0.90 (0.74 to 1.11)	0.326	0.750 (0.713 to 0.787)	0.170	0.05 (− 0.10 to 0.20)	0.495	0.001 (− 0.003 to 0.006)	0.606
+ Ventilatory ratio	0.99 (0.78 to 1.25)	0.938	0.749 (0.712 to 0.786)	0.170	− 0.00 (− 0.15 to 0.15)	0.996	0.000 (− 0.003 to 0.003)	0.994
+ end-tidal-to-arterial PCO_2_ ratio	0.87 (0.71 to 1.08)	0.206	0.743 (0.703 to 0.782)	0.170	− 0.09 (− 0.25 to 0.07)	0.257	− 0.006 (− 0.012 to − 0.000)	0.038

HB: Harris–Benedict; CI: confidence interval; AUC: area under the curve; NRI: net reclassification index; IDI: integrated discrimination index

*Represents the odds ratio for the lung-specific physiological variables in the multivariable model

All models are mixed-effect models with centers as random effect and considering a binomial distribution

All continuous variables were entered after standardization to improve convergence of the model, and odds ratio represent the increase in one standard deviation of the variable

### Sensitivity analysis

Results after multiple imputation were similar to the primary analysis (Additional File 1: Tables S8 and S9, and Figures S2 and S3).

### Post-hoc analysis

All analyses described above were repeated for Corrected Minute Ventilation and yielded similar results (Additional File 1: Tables S9, S10, S11, S12, S13, S14 and Figures S4 and S5). The change in measures of wasted ventilation between day 3 and day 0 of intubation were significantly associated with outcome (Additional File 1: Table S10). When restricting the analyses to patients with hypercapnia at presentation (PaCO_2_ > 50 mmHg) or prone positioning during the first day of mechanical ventilation, we did not observe large changes in effect sizes (Additional File 1: Table S12).

## Discussion

The findings of this multicenter, observational cohort study of COVID-19-related ARDS patients showed that estimates for dead space ventilation increased over the first days of invasive ventilation and were significantly higher in non-survivors than survivors. However, none of these indices was independently associated with mortality when corrected for potential confounders. Therefore, wasted ventilation, and, tentatively, increased estimated dead space fraction, may be a marker for disease severity rather than an independent predictor of outcome.

Despite [19] the potential clinical value, *V*_D_/*V*_T_ is not routinely measured in daily critical care practice. One possible barrier is the requirement of volumetric capnography (or other techniques of analyzing exhaled gas) to measure *V*_d_/*V*_t_. Estimated measures for calculating *V*_D_/*V*_T_ are more frequently utilized and a wide array of these indices were included in this study [6, 20]. VR is a recently validated index in patients under controlled modes of mechanical ventilation. This index was shown to be high in patients with COVID-19-related ARDS [3, 9] and is known to show moderate correlation with *V*_D_/*V*_T_ by volumetric capnography [7]. We found that the VR was not significantly different between survivors and non survivors at the start of ventilation and on day 1. However, we did find a significant difference in the following days of mechanical ventilation between survivors and non-survivors, not only for the VR but also for the rest of dead space estimates under study when a post-hoc analysis was performed (Table S14). This finding suggests the dynamic changes of these estimates over time are much more important than a single variable at the start of mechanical ventilation, also because this includes the response to optimization of ventilator settings.

Recently, the end-tidal-to-arterial PCO_2_ ratio (P_ET_CO_2_/PaCO_2_) has been described as another surrogate for *V*_D_/*V*_T_ in ARDS patients [15]. Each of these estimations has particular limitations, and they should be seen as complementary: if all point in the same direction, this likely reflects increased dead space ventilation. For example, in the presence of increased intrapulmonary shunt (as in ARDS patients), rising PaCO_2_ coincides with decreasing P_ET_CO2. Both shunt and low cardiac output states are known determinants of *V*_D_/*V*_T_. It is worth noting that that the impact of cardiac output exists only when measuring *V*_D_/*V*_T_ the Enghoff modification of Bohr's original formula is used. In the case of shunt, the increase in venous admixture will elevate the PaCO_2_ increasing dead space fraction [21]. This contribution is of special importance when *V*_D_/*V*_T_ is high, where physiologic dead space can be contaminated by the large shunt fractions present in any type of ARDS. In low cardiac output states, a decrease in pulmonary blood flow leads to a reduced alveolar CO_2_ delivery decreasing P_E_CO_2_, thereby increasing *V*_D_/*V*_T_ [22]. In both cases, indices for increase in dead space fraction would capture these phenomena and is hard to know each part's relative contribution in practice. Taken together, dead space indices reflect impaired outgassing of CO_2_ because of abnormal ventilation-perfusion matching giving a good global index of a lung’s gas exchange efficiency [23, 24].

Dead space estimations were significantly increased in non-survivors in the first four days of mechanical ventilation compared to survivors. This is line with previous studies in all patients with ARDS (not only those with COVID19), in which dead space (*V*_D_/*V*_T_) was elevated during the first week after start of invasive ventilation [4, 25]. We also described the association between these indices and outcome that was previously observed in patients with ARDS due to other causes than COVID-19 [4, 25]. However, in our study the investigated estimates did not add predictive value to a model that included other known predictors for 28-day mortality, with the possible exception of P_ET_CO_2_/PaCO_2_ at the start and at day 1 of ventilation. This contrasts with several studies in ARDS that showed increased dead space ventilation to be a robust and independent predictor of mortality risk [4, 25–27]. Decreasing P_ET_CO_2_/PaCO_2_ was also independently associated with mortality risk in one study [15]. Yet, our findings are in line with a previous report in which we assessed the added value of markers of impaired ventilation during the first days of mechanical ventilation in non-COVID-19-related ARDS [8]. Taken together, the data suggest that markers of impaired ventilation reflect disease severity but are not independent predictors of outcome, irrespective of the cause of ARDS.

Although not the primary aim of this study, we observed that patients who were deceased were less frequently put in prone positioning (Table S2). Prone position facilitates shape matching, which helps minimizing injurious ventilation and frequently improves gas exchange through better *V*/*Q* matching resulting in less shunt and improved CO_2_ clearance. Therefore, it could be postulated that prone positioning confounds the relation between surrogates of dead space ventilation and outcome. A post-hoc analysis, however, did not show a stronger association between these surrogates and outcomes in patients who did not receive prone position, yielding this explanation less likely.

In the current study in patients with COVID-19-related ARDS, impaired ventilation was already present in the first days of invasive ventilation. The studied estimations for *V*_D_/*V*_T_ further increased during the first days of invasive ventilation, especially in patients who did not survive. Altered hemostasis and thrombosis are postulated to be a key element of ARDS, with the endothelium playing a key role by promoting microthrombogenesis [28–30]. Endothelial infection and activation and disorders of the microvasculature have been described in the pathogenesis of COVID-19 [19], and perfusion defects in the pulmonary arterial circulation are frequently observed [31]. Autopsy findings include pulmonary vascular microthrombi [32] in addition to diffuse alveolar damage. These findings could lead to high dead space fraction.

The strengths of this study include the size of the multi-center cohort, careful data collection and with few missing data, the pre-specified analysis plan, and the evaluation of multiple estimations for impaired ventilation. The central limitation of this study is that we did not quantify dead space ventilation directly by volumetric capnography or another technique. This was not possible in the setting of a pandemic, where the critical care systems were overwhelmed with patients. A second limitation is the observational nature of the study. Therefore, this study does not provide insight into potential mechanisms that may contribute to the association between high dead space estimations and mortality in COVID-19-related ARDS patients. Another important aspect to take into account is the aspect of the instrumental dead space. Use of heated humidifiers of HMEs (heat and moisture exchangers) is heterogeneous in the clinical practice, and different HMEs have different dead space volumes. Instrumental dead space may significantly affect the total dead space, mainly when using low tidal volume ventilation, and we have commented on this previously [33].

For the estimated *V*_D_/*V*_T_ computed by the Harris–Benedict formula, we assumed an RQ of 0.8 for VCO_2_ calculation based on a previous study [6]. Although the RQ may vary among ARDS patients, a recently previous work showed a good correlation between the VR (which also depends on the VCO_2_) and dead space measured with volumetric capnography [7].

The results of this study indicate that estimations for increased dead space may not be independently associated with mortality. The observed effect sizes were remarkably similar to those observed in non-COVID-19-related ARDS. This contrasts several reports that have hypothesized that profound endothelial injury and coagulopathy may be central mediators of lung injury in COVID-19 [34]. We acknowledge that we did not measure these processes in this study, but we do provide evidence that COVID-19-related ARDS appears to be similar to non-COVID ARDS with respect to Vd/Vt. This implies that dead space and its estimates should be understood as a readily available marker of ARDS severity. Whether a high dead space identifies an enriched patient population with a higher prevalence of vascular injury, and who might benefit from treatments aimed at restoring normal pulmonary perfusion is unknown. Previous data suggest that some drugs with anticoagulant properties may decrease *V*_D_/*V*_T_ in patients with ARDS [35], making this an attractive hypothesis to consider.

At the moment, no data exists on the measurement of physiologic dead space in COVID-19-related ARDS by integrating volumetric capnography plots of eliminated CO_2_ concentration versus the respective expired tidal volume of a single breath. Since volumetric capnography offers a more in-depth representation of the kinetics of CO_2_ elimination per breath, the application of longitudinal or time-series models to analyze the effect of CO_2_ elimination impairment on outcome warrants further research.

## Conclusions

Estimates for wasted ventilation are abnormal at the start of invasive ventilation in patients with COVID-19-related ARDS and worsen during consequent days. Ventilation impairment seems to be more extensive in non-survivors than in survivors, but they may not yield prognostic information when added to a baseline risk model. In the absence of bedside capnography, dead space estimates may serve as an important tool to assess the severity of COVID-19-related ARDS along with other variables such as oxygenation abnormalities and respiratory mechanics.

## Supplementary information

**Additional file 1**. Impaired ventilation is not associated with 28-day mortality in COVID-19 ARDS

## Data Availability

Morales-Quinteros, Bos and Serpa Neto had full access to all data in the study and take responsibility for the integrity of the data and the accuracy of the data analysis; the members of the Steering Committee for PRoVENT–COVID Collaborative Group vouch for the accuracy and completeness of the data and for the fidelity of the study to the protocol.

## Abbreviations

ARDS: Acute respiratory distress syndrome
AUROCC: Area under the receiver operating characteristic curve
COVID-19: Coronavirus disease 2019
FiO_2_: Fraction of inspired oxygen
HB: Harris–Benedict
ICU: Intensive care unit
IBW: Ideal body weight
IDI: Integrated discrimination improvement
MERS: Middle East respiratory syndrome
PaCO_2_: Partial pressure of carbon dioxide in arterial blood
P_E_CO_2_: Expired partial pressure of carbon dioxide
PEEP: Positive end expiratory pressure
PaO_2_: Partial pressure of oxygen in arterial blood
PaO_2_/FiO_2_: Arterial oxygen tension to fraction of inspired oxygen
PRoVENT-COVID: PRactice of VENTilation in Patients with Coronavirus Disease 2019
SARS: Severe acute respiratory syndrome
V̇CO_2_: Carbon dioxide consumption
V̇_Ecorr_: Corrected minute ventilation
VR: Ventilatory ratio
V_D_/V_T_: Dead space fraction
