# Supplementary material for: Dead space estimates may not be independently associated with 28-day mortality in COVID-19 ARDS
Source: Crit Care. 2021 May 17;25:171. doi: 10.1186/s13054-021-03570-0 (PMC8127435; doi:10.1186/s13054-021-03570-0)

**Dead space estimates may not be independently associated with 28-day mortality in COVID-19 ARDS**

ONLINE SUPPLEMENT

**eMETHODS**

*Direct estimation of dead space fraction*

This estimate is an alternative approach to estimate dead space fraction directly through anthropometric and respiratory variables such as respiratory rate, minute ventilation, PaCO_2_, PEEP, and the Murray lung injury score using the Eq. (1):

$$\frac{V_{D}}{V_{T}}=0.1726+\left( 0.0059*RR \right)+\left( 0.0054*PEEP \right)+\left( 0.0293*LIS \right)+\left( 0.0036*{PaCO}_{2}* V_{E} \right)+\left( 0.000057* {PaCO}_{2}*age \right) Eq. (1)$$

RR is the respiratory rate in breaths per minute, PEEP the positive end-expiratory pressure in cmH_2_O, LIS is the Murray lung injury score,^1^ PaCO_2_ is the partial pressure of carbon dioxide in mmHg, and V_E_ is the minute ventilation in liters/minute.

*End-tidal-to-arterial PCO_2_ ratio*

End-tidal-to-arterial PCO_2_ ratio was calculated as previously described using the Eq. (2):^2^

$$\frac{P_{ET}{CO}_{2}}{{PaCO}_{2}} Eq. (2)$$

*Multiple imputation*

An additional model considering a multiple imputation by chained equation method (MICE) was conducted. The following variables were considered in the multiple imputation process: age, gender, body mass index, PaO_2_ / FiO_2_ ratio, creatinine, hypertension, diabetes, use of angiotensin converting enzyme inhibitors (ACEi), use of angiotensin II receptor blockers (ARB), use of vasopressor or inotropic, fluid balance, pH, mean arterial pressure, heart rate, tidal volume, peak pressure, PEEP, respiratory system compliance, and 28-day mortality.

Since respiratory system compliance, tidal volume, peak pressure and PEEP are passive variables, the method ‘Just Another Variable’ was used, imputing the source variables (tidal volume, peak pressure and PEEP) and also the derived variable (respiratory system compliance) separately. It is expected that the ‘Just Another Variable’ approach will make a very bad approximation to the joint density of the variables; however, it can yield valid inferences for the analysis model.^3,4^

The lower and upper cut-offs of imputed values respected the upper and lower limits of the original variables in patients without missing. Imputation was performed using predictive mean matching for continuous variables, logistic regression for binary variables and polytomous regression for categorical variables with more than two categories. A model with five imputed datasets with 50 iterations was used. After imputation all models were repeated and results combined across imputations using Rubin’s rule.

**References**

1. Murray JF, Matthay MA, Luce JM, Flick MR. An expanded definition of the adult respiratory distress syndrome. Am Rev Respir Dis 1988; 138:720-3.

2. Kallet RH, Lipnick MS. End-tidal-to-arterial PCO2 ratio as signifier for physiologic deadspace ratio and oxygenation dysfunction in acute respiratory distress syndrome. Respir Care 2020;[in press].

3. White IR, Royston P, Wood AM (2011) Multiple imputation using chained equations: Issues and guidance for practice. Stat Med 30:377-99

4. Von Hippel PT (2009) How to impute squares, interactions, and other transformed variables. Sociological Methodology 39:265-91.

| **eTable 1 - Amount of Missing Data** | | | |
| --- | --- | --- | --- |
|  | **All Patients**  **(*n* = 927)** | **Non-Survivors**  **(*n* = 266)** | **Survivors**  **(*n* = 661)** |
| Age | 0 (0.0) | 0 (0.0) | 0 (0.0) |
| Gender | 0 (0.0) | 0 (0.0) | 0 (0.0) |
| Body mass index | 2 (0.2) | 0 (0.0) | 2 (0.3) |
| Transferred intubated | 0 (0.0) | 0 (0.0) | 0 (0.0) |
| Use of non-invasive ventilation | 58 (6.3) | 15 (5.6) | 43 (6.5) |
| Chest CT scan performed | 14 (1.5) | 2 (0.8) | 12 (1.8) |
| Chest X-ray performed | 332 (35.8) | 105 (39.5) | 227 (34.3) |
| SAPS II | 599 (64.6) | 176 (66.2) | 423 (64.0) |
| APACHE II | 687 (74.1) | 190 (71.4) | 497 (75.2) |
| APACHE IV | 527 (56.9) | 150 (56.4) | 377 (57.0) |
| SOFA | 474 (51.1) | 142 (53.4) | 332 (50.2) |
| Severity ARDS | 6 (0.6) | 2 (0.8) | 4 (0.6) |
| Hypertension | 0 (0.0) | 0 (0.0) | 0 (0.0) |
| Heart failure | 0 (0.0) | 0 (0.0) | 0 (0.0) |
| Diabetes | 0 (0.0) | 0 (0.0) | 0 (0.0) |
| Chronic kidney disease | 0 (0.0) | 0 (0.0) | 0 (0.0) |
| Baseline creatinine | 24 (2.6) | 8 (3.0) | 16 (2.4) |
| Liver cirrhosis | 0 (0.0) | 0 (0.0) | 0 (0.0) |
| Chronic obstructive pulmonary disease | 0 (0.0) | 0 (0.0) | 0 (0.0) |
| Active hematological neoplasia | 0 (0.0) | 0 (0.0) | 0 (0.0) |
| Active solid neoplasia | 0 (0.0) | 0 (0.0) | 0 (0.0) |
| Neuromuscular disease | 0 (0.0) | 0 (0.0) | 0 (0.0) |
| Immunosuppression | 0 (0.0) | 0 (0.0) | 0 (0.0) |
| Systemic steroids | 0 (0.0) | 0 (0.0) | 0 (0.0) |
| Inhaled steroids | 0 (0.0) | 0 (0.0) | 0 (0.0) |
| Angiotensin converting enzyme inhibitor | 0 (0.0) | 0 (0.0) | 0 (0.0) |
| Angiotensin II receptor blocker | 0 (0.0) | 0 (0.0) | 0 (0.0) |
| Beta-blockers | 0 (0.0) | 0 (0.0) | 0 (0.0) |
| Insulin | 0 (0.0) | 0 (0.0) | 0 (0.0) |
| Metformin | 0 (0.0) | 0 (0.0) | 0 (0.0) |
| Statins | 0 (0.0) | 0 (0.0) | 0 (0.0) |
| Calcium channel blockers | 0 (0.0) | 0 (0.0) | 0 (0.0) |
| Fluid balance | 15 (1.6) | 5 (1.9) | 10 (1.5) |
| Urine output | 17 (1.8) | 7 (2.6) | 10 (1.5) |
| Recruitment maneuver | 162 (17.5) | 39 (14.7) | 123 (18.6) |
| Prone positioning | 7 (0.8) | 1 (0.4) | 6 (0.9) |
| Use of neuromuscular blocking agent | 0 (0.0) | 0 (0.0) | 0 (0.0) |
| Extracorporeal membrane oxygenation | 13 (1.4) | 2 (0.8) | 11 (1.7) |
| Use of continuous sedation | 0 (0.0) | 0 (0.0) | 0 (0.0) |
| Use of vasopressor | 0 (0.0) | 0 (0.0) | 0 (0.0) |
| Use of inotropic drugs | 0 (0.0) | 0 (0.0) | 0 (0.0) |
| Ventilator-free days at day 28 | 36 (3.9) | 0 (0.0) | 36 (5.4) |
| Successful extubation | 5 (0.5) | 1 (0.4) | 4 (0.6) |
| Duration of ventilation | 37 (4.0) | 1 (0.4) | 36 (5.4) |
| Tracheostomy | 8 (0.9) | 0 (0.0) | 8 (1.2) |
| Reintubation | 12 (1.3) | 2 (0.8) | 10 (1.5) |
| Pneumothorax | 34 (3.7) | 8 (3.0) | 26 (3.9) |
| Thromboembolic complications | 0 (0.0) | 0 (0.0) | 0 (0.0) |
| Acute kidney injury | 4 (0.4) | 0 (0.0) | 4 (0.6) |
| Need for renal replacement therapy | 0 (0.0) | 0 (0.0) | 0 (0.0) |
| ICU length of stay | 27 (2.9) | 2 (0.8) | 25 (3.8) |
| Hospital length of stay | 56 (6.0) | 0 (0.0) | 56 (8.5) |
| ICU mortality | 14 (1.5) | 2 (0.8) | 12 (1.8) |
| Hospital mortality | 61 (6.6) | 3 (1.1) | 58 (8.8) |
| 28-Day mortality | 0 (0.0) | 0 (0.0) | 0 (0.0) |
| 90-Day mortality | 62 (6.7) | 0 (0.0) | 62 (9.4) |
| Mode of ventilation | 49 (5.3) | 16 (6.0) | 33 (5.0) |
| Tidal volume | 0 (0.0) | 0 (0.0) | 0 (0.0) |
| PEEP | 3 (0.3) | 1 (0.4) | 2 (0.3) |
| Peak pressure | 74 (8.0) | 29 (10.9) | 45 (6.8) |
| Driving pressure | 76 (8.2) | 29 (10.9) | 47 (7.1) |
| Compliance | 76 (8.2) | 29 (10.9) | 47 (7.1) |
| Total respiratory rate | 0 (0.0) | 0 (0.0) | 0 (0.0) |
| FiO_2_ | 5 (0.5) | 2 (0.8) | 3 (0.5) |
| SpO_2_ | 2 (0.2) | 0 (0.0) | 2 (0.3) |
| etCO_2_ | 67 (7.2) | 27 (10.2) | 40 (6.1) |
| pH | 0 (0.0) | 0 (0.0) | 0 (0.0) |
| PaO_2_ | 0 (0.0) | 0 (0.0) | 0 (0.0) |
| PaO_2_ / FiO_2_ | 6 (0.6) | 2 (0.8) | 4 (0.6) |
| PaCO_2_, mmHg | 0 (0.0) | 0 (0.0) | 0 (0.0) |
| Lactate, mmol/L | 113 (12.2) | 26 (9.8) | 87 (13.2) |
| Heart rate | 1 (0.1) | 1 (0.4) | 0 (0.0) |
| Mean arterial pressure | 3 (0.3) | 1 (0.4) | 2 (0.3) |

| **eTable 2 - Ventilatory Variables at Start of Ventilation** | | | | |
| --- | --- | --- | --- | --- |
|  | **All Patients**  **(*n* = 927)** | **Non-Survivors**  **(*n* = 266)** | **Survivors**  **(*n* = 661)** | ***p* value** |
| Mode of ventilation – no (%) |  |  |  | 0.356 |
| Volume controlled | 152 (16.5) | 53 (19.9) | 99 (15.1) |  |
| Pressure controlled | 513 (55.6) | 135 (50.8) | 378 (57.5) |  |
| Pressure support | 26 (2.8) | 5 (1.9) | 21 (3.2) |  |
| SIMV | 75 (8.1) | 24 (9.0) | 51 (7.8) |  |
| APRV | 29 (3.1) | 10 (3.8) | 19 (2.9) |  |
| INTELLiVENT-ASV | 40 (4.3) | 12 (4.5) | 28 (4.3) |  |
| Other | 88 (9.5) | 27 (10.2) | 61 (9.3) |  |
| Assisted ventilation – no (%) | 258 (28.0) | 78 (29.3) | 180 (27.4) | 0.571 |
| Total respiratory rate, mpm | 21.5 (19.3 – 24.0) | 21.4 (19.3 – 24.0) | 21.5 (19.3 – 24.0) | 0.798 |
| FiO_2_ | 0.57 (0.50 – 0.68) | 0.60 (0.50 – 0.70) | 0.57 (0.49 – 0.67) | 0.006 |
| Laboratory tests |  |  |  |  |
| pH | 7.36 (7.32 – 7.41) | 7.34 (7.29 – 7.39) | 7.37 (7.33 – 7.41) | < 0.001 |
| PaO_2_, mmHg | 81.8 (72.8 – 96.4) | 83.0 (74.3 – 95.2) | 81.5 (72.5 – 97.0) | 0.403 |
| PaO_2_ / FiO_2_ | 130.9 (99.9 – 175.5) | 137.2 (98.1 – 180.0) | 128.8 (100.2 – 171.5) | 0.262 |
| PaCO_2_, mmHg | 44.0 (39.1 – 49.5) | 44.9 (39.5 – 51.0) | 43.9 (39.0 – 49.1) | 0.106 |
| Lactate, mmol/L | 1.1 (0.9 – 1.4) | 1.3 (1.0 – 1.6) | 1.1 (0.9 – 1.4) | < 0.001 |
| Creatinine, µmol/L | 73.0 (61.0 – 95.5) | 83.5 (65.0 – 118.5) | 71.0 (59.0 – 89.0) | < 0.001 |
| Rescue therapy – no (%) |  |  |  |  |
| Prone positioning | 278 (30.4) | 65 (24.6) | 213 (32.8) | 0.017 |
| Duration, hours^a^ | 8.0 (4.0 – 14.0) | 7.0 (2.8 – 13.0) | 9.0 (5.0 – 14.0) | 0.050 |
| Recruitment maneuver | 18 (2.4) | 6 (2.7) | 12 (2.3) | 0.796 |
| ECMO | 0 (0.0) | 0 (0.0) | 0 (0.0) | 0.999 |
| Use of NMBA | 242 (26.2) | 70 (26.4) | 172 (26.1) | 0.934 |
| Hours of use of use^a^ | 0.0 (0.0 – 8.0) | 0.0 (0.0 – 8.0) | 0.0 (0.0 – 8.0) | 0.945 |
| Data are median (quartile 25% - quartile 75%) or No (%). Percentages may not total 100 because of rounding  *APRV: airway pressure release ventilation; ASV: adaptive support ventilation; ECMO: extracorporeal membrane oxygenation; FiO_2_: inspired fraction of oxygen; PCV: pressure-controlled ventilation; PEEP: positive end-expiratory pressure; PSV: pressure support ventilation; SIMV: synchronized intermittent mandatory ventilation; VCV: volume-controlled ventilation; NMBA: neuromuscular blocking agent*  ^a^ in patients who received it | | | | |

| **eTable 3 - Clinical Outcomes in the Included Population** | |
| --- | --- |
|  | **All Patients**  **(*n* = 927)** |
| Ventilator-free days at day 28, days | 3.0 (0.0 – 17.0) |
| Duration of ventilation, days | 13.0 (8.0 – 23.0) |
| In survivors at day 28, days | 15.0 (9.0 – 27.0) |
| Tracheostomy – no (%) | 140 (15.2) |
| Reintubation – no (%) | 114 (12.5) |
| Pneumothorax – no (%) | 6 (0.7) |
| Thromboembolic complications – no (%) | 267 (28.8) |
| Pulmonary embolism | 210 (22.7) |
| Deep vein thrombosis | 47 (5.1) |
| Ischemic stroke | 27 (2.9) |
| Myocardial infarction | 13 (1.4) |
| Systemic arterial embolism | 4 (0.4) |
| Acute kidney injury – no (%) | 406 (44.0) |
| Need for RRT – no (%) | 157 (16.9) |
| Need of rescue therapy – no (%)* | 686 (74.8) |
| Prone positioning | 529 (57.5) |
| Recruitment maneuver | 48 (6.3) |
| Use of NMBA | 445 (48.0) |
| ECMO | 8 (0.9) |
| Use of continuous sedation – no (%)* | 920 (99.2) |
| Use of inotropic or vasopressor – no (%)* | 876 (94.5) |
| Use of vasopressor | 875 (94.4) |
| Use of inotropic | 93 (10.0) |
| ICU length of stay, days | 15.0 (9.0 – 26.3) |
| In survivors, days | 18.0 (11.0 – 30.0) |
| Hospital length of stay, days | 24.0 (14.0 – 37.0) |
| In survivors, days | 30.0 (20.0 – 44.0) |
| ICU mortality – no (%) | 292 (32.0) |
| Hospital mortality – no (%) | 300 (34.6) |
| 28-day mortality – no (%) | 266 (28.7) |
| 90-day mortality – no (%) | 317 (36.6) |
| Data are median (quartile 25% - quartile 75%) or No (%). Percentages may not total 100 because of rounding  *RRT: renal replacement therapy; NMBA: neuromuscular blocking agent; ECMO: extracorporeal membrane oxygenation; ICU: intensive care unit; PEEP positive end expiratory pressure*  * assessed in the first four days of ventilation | |

| **eTable 4 - Univariable Effect of Lung-Specific Physiological Variables and Covariates on 28-Day Mortality** | | |
| --- | --- | --- |
|  | **Odds Ratio**  **(95% CI)** | ***p* value** |
| Lung-specific physiological variables at start of ventilation |  |  |
| Dead space fraction by Harris-Benedict | 10.57 (2.65 to 42.20) | 0.001 |
| Dead space fraction by direct estimate | 1.33 (1.03 to 1.73) | 0.031 |
| Ventilatory ratio | 1.20 (0.94 to 1.52) | 0.135 |
| end-tidal-to-arterial PCO_2_ ratio | 0.16 (0.06 to 0.42) | < 0.001 |
| Lung-specific physiological variables on day 01 |  |  |
| Dead space fraction by Harris-Benedict | 19.33 (3.99 to 93.51) | < 0.001 |
| Dead space fraction by direct estimate | 1.29 (0.97 to 1.70) | 0.076 |
| Ventilatory ratio | 1.12 (0.89 to 1.41) | 0.337 |
| end-tidal-to-arterial PCO_2_ ratio | 0.08 (0.02 to 0.27) | < 0.001 |
| Demographic characteristics |  |  |
| Age | 1.08 (1.06 to 1.10) | < 0.001 |
| Male gender | 1.46 (1.04 to 2.05) | 0.029 |
| Body mass index | 0.98 (0.95 to 1.01) | 0.198 |
| Co-existing disorders |  |  |
| Hypertension | 1.62 (1.19 to 2.19) | 0.002 |
| Diabetes | 1.78 (1.27 to 2.50) | 0.001 |
| Use of angiotensin converting enzyme inhibitors | 1.37 (0.94 to 1.99) | 0.097 |
| Use of angiotensin II receptor blocker | 1.31 (0.84 to 2.06) | 0.232 |
| Laboratory on the first day of ventilation |  |  |
| PaO_2_ / FiO_2_ | 1.00 (1.00 to 1.00) | 0.107 |
| Baseline creatinine | 1.00 (1.00 to 1.00) | 0.241 |
| pH | 0.00 (0.00 to 0.02) | < 0.001 |
| Organ supports at start of ventilation |  |  |
| Use of inotropic or vasopressors | 1.16 (0.80 to 1.69) | 0.421 |
| Signs at start of ventilation |  |  |
| Fluid balance | 1.00 (1.00 to 1.00) | 0.122 |
| Mean arterial pressure | 1.01 (1.00 to 1.02) | 0.015 |
| Heart rate | 0.98 (0.97 to 1.00) | 0.026 |
| Ventilatory variables at start of ventilation |  |  |
| Respiratory system compliance | 1.00 (0.99 to 1.02) | 0.716 |
| Positive end-expiratory pressure | 1.05 (0.98 to 1.11) | 0.139 |
| All models are mixed-effect models with centers as random effect and considering a binomial distribution.  All odds ratios represent an 1-point increase in continuous predictors | | |

| **eTable 5 - Multivariable Baseline Risk Model** | | |
| --- | --- | --- |
|  | **Odds Ratio**  **(95% CI)** | ***p* value** |
| Demographic characteristics |  |  |
| Age | 2.12 (1.70 to 2.64) | < 0.001 |
| Male gender | 1.43 (0.95 to 2.14) | 0.084 |
| Body mass index | 1.04 (0.83 to 1.30) | 0.728 |
| Co-existing disorders |  |  |
| Hypertension | 1.34 (0.91 to 1.99) | 0.143 |
| Diabetes | 1.22 (0.82 to 1.81) | 0.328 |
| Use of angiotensin converting enzyme inhibitors | 0.94 (0.59 to 1.50) | 0.803 |
| Use of angiotensin II receptor blocker | 0.78 (0.44 to 1.37) | 0.387 |
| Laboratory at start of ventilation |  |  |
| PaO_2_ / FiO_2_ | 0.97 (0.81 to 1.17) | 0.751 |
| Baseline creatinine | 1.00 (0.85 to 1.18) | 0.958 |
| pH | 0.76 (0.63 to 0.91) | 0.003 |
| Organ supports at start of ventilation |  |  |
| Use of inotropic or vasopressors | 0.99 (0.64 to 1.52) | 0.947 |
| Signs at start of ventilation |  |  |
| Fluid balance | 0.98 (0.82 to 1.16) | 0.810 |
| Mean arterial pressure | 0.87 (0.73 to 1.03) | 0.108 |
| Heart rate | 1.23 (1.02 to 1.47) | 0.026 |
| Ventilatory variables at start of ventilation |  |  |
| Respiratory system compliance | 1.00 (0.84 to 1.20) | 0.965 |
| Positive end-expiratory pressure | 1.16 (0.97 to 1.39) | 0.097 |
| All models are mixed-effect models with centers as random effect and considering a binomial distribution  All continuous variables were entered after standardization to improve convergence of the model, and odds ratio represent the increase in one standard deviation of the variable. | | |

| **eTable 6 - Assessment of Multicollinearity in the Multivariable Baseline Risk Model** | |
| --- | --- |
|  | **Variance Inflation Factor** |
| Demographic characteristics |  |
| Age | 1.132540 |
| Male gender | 1.148757 |
| Body mass index | 1.245690 |
| Co-existing disorders |  |
| Hypertension | 1.366087 |
| Diabetes | 1.108523 |
| Use of angiotensin converting enzyme inhibitors | 1.250193 |
| Use of angiotensin II receptor blocker | 1.240720 |
| Laboratory at start of ventilation |  |
| PaO_2_ / FiO_2_ | 1.098762 |
| Baseline creatinine | 1.095174 |
| pH | 1.299089 |
| Organ supports at start of ventilation |  |
| Use of inotropic or vasopressors | 1.090880 |
| Signs at start of ventilation |  |
| Fluid balance | 1.104375 |
| Mean arterial pressure | 1.084226 |
| Heart rate | 1.278828 |
| Ventilatory variables at start of ventilation |  |
| Respiratory system compliance | 1.190230 |
| Positive end-expiratory pressure | 1.109731 |
| All models are mixed-effect models with centers as random effect and considering a binomial distribution  All continuous variables were entered after standardization to improve convergence of the model, and odds ratio represent the increase in one standard deviation of the variable. | |

| **eTable 7 - Assessment of Linearity Assumption in the Multivariable Baseline Risk Model** | |
| --- | --- |
|  | ***p* value** |
| Demographic characteristics |  |
| Age | 0.679 |
| Body mass index | 0.744 |
| Laboratory at start of ventilation |  |
| PaO_2_ / FiO_2_ | 0.730 |
| Baseline creatinine | 0.494 |
| pH | 0.848 |
| Signs at start of ventilation |  |
| Mean arterial pressure | 0.613 |
| Heart rate | 0.373 |
| Ventilatory variables at start of ventilation |  |
| Respiratory system compliance | 0.919 |
| Positive end-expiratory pressure | 0.801 |
| Linearity assumption assessed using the Box-Tidwell power transformation | |

| **eTable 8 - Multivariable Baseline Risk Model After Multiple Imputation** | | |
| --- | --- | --- |
|  | **Odds Ratio**  **(95% CI)** | ***p* value** |
| Demographic characteristics |  |  |
| Age | 2.19 (1.74 to 2.75) | < 0.001 |
| Male gender | 1.36 (0.90 to 2.06) | 0.144 |
| Body mass index | 1.02 (0.81 to 1.29) | 0.841 |
| Co-existing disorders |  |  |
| Hypertension | 1.37 (0.91 to 2.05) | 0.133 |
| Diabetes | 1.20 (0.80 to 1.80) | 0.385 |
| Use of angiotensin converting enzyme inhibitors | 0.94 (0.58 to 1.51) | 0.785 |
| Use of angiotensin II receptor blocker | 0.77 (0.43 to 1.37) | 0.367 |
| Laboratory at start of ventilation |  |  |
| PaO_2_ / FiO_2_ | 0.92 (0.76 to 1.11) | 0.380 |
| Baseline creatinine | 1.01 (0.86 to 1.19) | 0.923 |
| pH | 0.77 (0.63 to 0.93) | 0.006 |
| Organ supports at start of ventilation |  |  |
| Use of inotropic or vasopressors | 1.09 (0.69 to 1.72) | 0.727 |
| Signs at start of ventilation |  |  |
| Fluid balance | 0.99 (0.83 to 1.18) | 0.896 |
| Mean arterial pressure | 0.87 (0.73 to 1.04) | 0.134 |
| Heart rate | 1.22 (1.01 to 1.48) | 0.035 |
| Ventilatory variables at start of ventilation |  |  |
| Respiratory system compliance | 1.01 (0.84 to 1.22) | 0.906 |
| Positive end-expiratory pressure | 1.14 (0.94 to 1.37) | 0.178 |
| All models are mixed-effect models with centers as random effect and considering a binomial distribution  All continuous variables were entered after standardization to improve convergence of the model, and odds ratio represent the increase in one standard deviation of the variable. | | |

| **eTable 9 - Predictive Accuracy of Lung-Specific Physiological Variables After Multiple Imputation** | | | | |
| --- | --- | --- | --- | --- |
|  | **Odds Ratio***  **(95% CI)** | ***p* value** | **AUC**  **(95% CI)** | **Brier Score** |
| Base model | --- | --- | 0.732 (0.601 to 0.832) | 0.175 |
| At start of ventilation |  |  |  |  |
| + Dead space fraction by HB | 0.90 (0.73 to 1.11) | 0.315 | 0.732 (0.601 to 0.832) | 0.175 |
| + Dead space fraction direct | 0.82 (0.66 to 1.02) | 0.069 | 0.733 (0.602 to 0.833) | 0.175 |
| + Ventilatory ratio | 0.89 (0.70 to 1.14) | 0.376 | 0.732 (0.601 to 0.832) | 0.175 |
| + end-tidal-to-arterial PCO_2_ ratio | 0.98 (0.79 to 1.21) | 0.838 | 0.732 (0.602 to 0.832) | 0.175 |
| Day 01 |  |  |  |  |
| + Dead space fraction by HB | 1.00 (0.81 to 1.23) | 0.987 | 0.732 (0.601 to 0.832) | 0.175 |
| + Dead space fraction direct | 0.86 (0.70 to 1.06) | 0.169 | 0.732 (0.601 to 0.832) | 0.175 |
| + Ventilatory ratio | 0.94 (0.74 to 1.20) | 0.609 | 0.732 (0.601 to 0.832) | 0.175 |
| + end-tidal-to-arterial PCO_2_ ratio | 0.94 (0.76 to 1.17) | 0.581 | 0.734 (0.604 to 0.833) | 0.175 |
| *HB: Harris-Benedict; CI: confidence interval; AUC: area under the curve; NRI: net reclassification index; IDI: integrated discrimination index*  * represents the odds ratio for the lung-specific physiological variables in the multivariable model  All models are mixed-effect models with centers as random effect and considering a binomial distribution  All continuous variables were entered after standardization to improve convergence of the model, and odds ratio represent the increase in one standard deviation of the variable. | | | | |

| **eTable 10 - Lung-Specific Physiological Variables in the First Four Days of Ventilation According to 28-Day Mortality** | | | | | | | |
| --- | --- | --- | --- | --- | --- | --- | --- |
|  | **All Patients**  **(*n* = 927)** | **Non-Survivors**  **(*n* = 266)** | | **Survivors**  **(*n* = 661)** | | ***p* value*** | |
| V̇Ecorr |  |  | |  | |  | |
| At start of ventilation | 11.11 ± 3.51 | 11.43 ± 3.89 | | 10.98 ± 3.34 | | 0.093 | |
| Day 01 | 11.96 ± 3.49 | 12.18 ± 3.54 | | 11.87 ± 3.46 | | 0.119 | |
| Day 02 | 12.85 ± 3.71 | 13.51 ± 4.09 | | 12.58 ± 3.51 | | < 0.001 | |
| Day 03 | 13.72 ± 4.19 | 14.62 ± 4.64 | | 13.37 ± 3.95 | | < 0.001 | |
| ***p* value (interaction survival x day)** |  | < 0.001 | | | |  | |
| Data are median (quartile 25% - quartile 75%)  *HB: Harris-Benedict*  * calculated using pairwise contrasts in a mixed-effect generalized linear model considering a Gaussian distribution and with day, group and an interaction day x group as fixed effect, and with patients and center as random effect. A binomial distribution was used for binary variables and a Gaussian distribution for continuous | | | | | | | |
| **eTable 11 - Univariable Effect of Lung-Specific Physiological Variables and Covariates on 28-Day Mortality** | | | | | | |  |
|  | | | **Odds Ratio**  **(95% CI)** | | ***p* value** | |  |
| Lung-specific physiological variables at start of ventilation | | |  | |  | |  |
| V̇Ecorr | | | 1.03 (0.99 to 1.08) | | 0.112 | |  |
| Lung-specific physiological variables on day 01 | | |  | |  | |  |
| V̇Ecorr | | | 1.03 (0.98 to 1.07) | | 0.208 | |  |
| All models are mixed-effect models with centers as random effect and considering a binomial distribution.  All odds ratios represent an 1-point increase in continuous predictors | | | | | | |  |

| **eTable 12 - Univariable Effect of Lung-Specific Physiological Variables and Covariates on 28-Day Mortality (Only Patients with Baseline PaCO_2_ ≥ 50 mmHg; n = 214 patients)** | | |
| --- | --- | --- |
|  | **Odds Ratio**  **(95% CI)** | ***p* value** |
| Lung-specific physiological variables at start of ventilation |  |  |
| Dead space fraction by Harris-Benedict | 61.83 (2.03 to 1885.84) | 0.018 |
| Dead space fraction by direct estimate | 1.49 (0.97 to 2.31) | 0.072 |
| Ventilatory ratio | 1.17 (0.85 to 1.61) | 0.329 |
| end-tidal-to-arterial PCO_2_ ratio | 0.15 (0.02 to 1.12) | 0.065 |
| V̇Ecorr | 1.06 (0.99 to 1.13) | 0.080 |
| Lung-specific physiological variables on day 01 |  |  |
| Dead space fraction by Harris-Benedict | 33.49 (0.71 to 1572.12) | 0.074 |
| Dead space fraction by direct estimate | 1.38 (0.84 to 2.27) | 0.198 |
| Ventilatory ratio | 1.07 (0.80 to 1.44) | 0.632 |
| end-tidal-to-arterial PCO_2_ ratio | 0.33 (0.04 to 3.19) | 0.341 |
| V̇Ecorr | 1.05 (0.97 to 1.13) | 0.232 |
| All models are mixed-effect models with centers as random effect and considering a binomial distribution.  All odds ratios represent an 1-point increase in continuous predictors | | |

| **eTable 13 - Univariable Effect of Lung-Specific Physiological Variables and Covariates on 28-Day Mortality (Only Patients not Receiving Prone at Baseline; n = 635 patients)** | | |
| --- | --- | --- |
|  | **Odds Ratio**  **(95% CI)** | ***p* value** |
| Lung-specific physiological variables at start of ventilation |  |  |
| Dead space fraction by Harris-Benedict | 12.55 (2.31 to 68.13) | 0.003 |
| Dead space fraction by direct estimate | 1.48 (1.03 to 2.14) | 0.034 |
| Ventilatory ratio | 1.19 (0.88 to 1.60) | 0.252 |
| end-tidal-to-arterial PCO_2_ ratio | 0.08 (0.03 to 0.26) | < 0.001 |
| V̇Ecorr | 1.04 (0.99 to 1.11) | 0.132 |
| Lung-specific physiological variables on day 01 |  |  |
| Dead space fraction by Harris-Benedict | 14.40 (2.19 to 94.79) | 0.006 |
| Dead space fraction by direct estimate | 1.21 (0.85 to 1.71) | 0.290 |
| Ventilatory ratio | 1.03 (0.79 to 1.34) | 0.829 |
| end-tidal-to-arterial PCO_2_ ratio | 0.04 (0.01 to 0.17) | < 0.001 |
| V̇Ecorr | 1.02 (0.96 to 1.07) | 0.507 |
| All models are mixed-effect models with centers as random effect and considering a binomial distribution.  All odds ratios represent an 1-point increase in continuous predictors | | |

| **eTable 14 - Univariable Effect of Change in Lung-Specific Physiological Variables and Covariates on 28-Day Mortality** | | |
| --- | --- | --- |
|  | **Odds Ratio**  **(95% CI)** | ***p* value** |
| Change in lung-specific physiological variables (D3 - D0) |  |  |
| Dead space fraction by Harris-Benedict | 4.47 (0.83 to 23.91) | 0.080 |
| Dead space fraction by direct estimate | 1.50 (1.13 to 1.99) | 0.005 |
| Ventilatory ratio | 1.55 (1.16 to 2.08) | 0.003 |
| end-tidal-to-arterial PCO_2_ ratio | 0.52 (0.19 to 1.38) | 0.186 |
| V̇Ecorr | 1.07 (1.02 to 1.11) | 0.005 |
| All models are mixed-effect models with centers as random effect and considering a binomial distribution.  All odds ratios represent an 1-point increase in continuous predictors | | |

| **eTable 15 – Radiographic findings of the Patients According to 28-Day Mortality** | | | | |
| --- | --- | --- | --- | --- |
|  | **All Patients**  **(*n* = 927)** | **Non-Survivors**  **(*n* = 266)** | **Survivors**  **(*n* = 661)** | ***p* value** |
| Chest CT scan performed – no (%) | 320 / 913 (35.0) | 102 / 264 (38.6) | 218 / 649 (33.6) | 0.145 |
| Lung parenchyma affected – no (%) |  |  |  | 0.659 |
| 0% | 13 / 320 (4.1) | 3 / 102 (2.9) | 10 / 218 (4.6) |  |
| 25% | 103 / 320 (32.2) | 31 / 102 (30.4) | 72 / 218 (33.0) |  |
| 50% | 95 / 320 (29.7) | 33 / 102 (32.4) | 62 / 218 (28.4) |  |
| 75% | 91 / 320 (28.4) | 27 / 102 (26.5) | 64 / 218 (29.4) |  |
| 100% | 18 / 320 (5.6) | 8 / 102 (7.8) | 10 / 218 (4.6) |  |
| Chest X-ray performed – no (%) | 526 / 595 (88.4) | 139 / 161 (86.3) | 387 / 434 (89.2) | 0.387 |
| Quadrants affected – no (%) |  |  |  | 0.286 |
| 1 | 40 / 525 (7.6) | 10 / 142 (7.0) | 30 / 383 (7.8) |  |
| 2 | 125 / 525 (23.8) | 28 / 142 (19.7) | 97 / 383 (25.3) |  |
| 3 | 147 / 525 (28.0) | 48 / 142 (33.8) | 99 / 383 (25.8) |  |
| 4 | 213 / 525 (40.6) | 56 / 142 (39.4) | 157 / 383 (41.0) |  |
| Data are median (quartile 25% - quartile 75%) or No (%). Percentages may not total 100 because of rounding  *CT: computed tomography* | | | | |

**eFigure 1 - Flowchart of Inclusions**


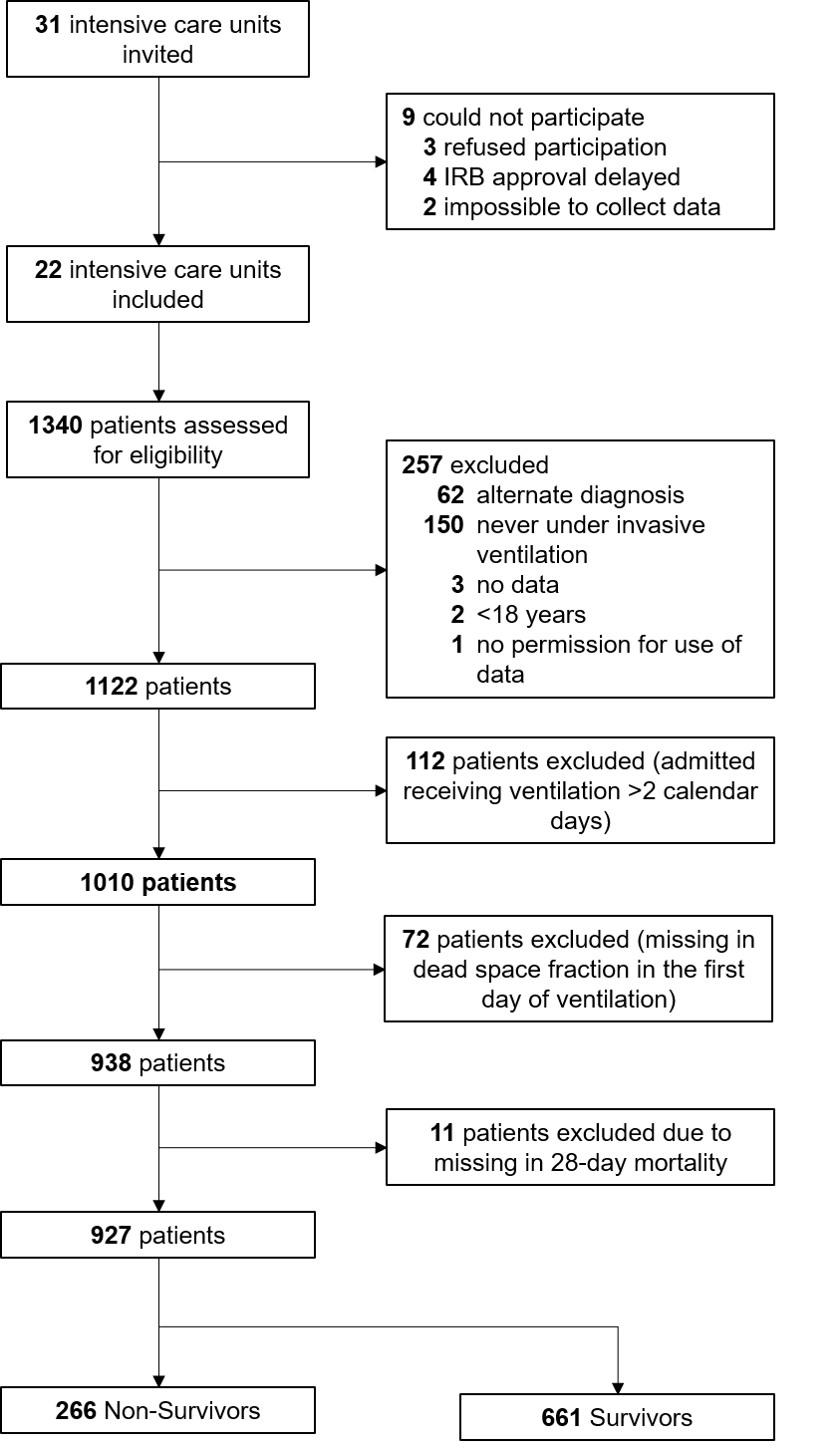


**eFigure 2 - Density Plot of Continuous Variables After Multiple Imputation**

**
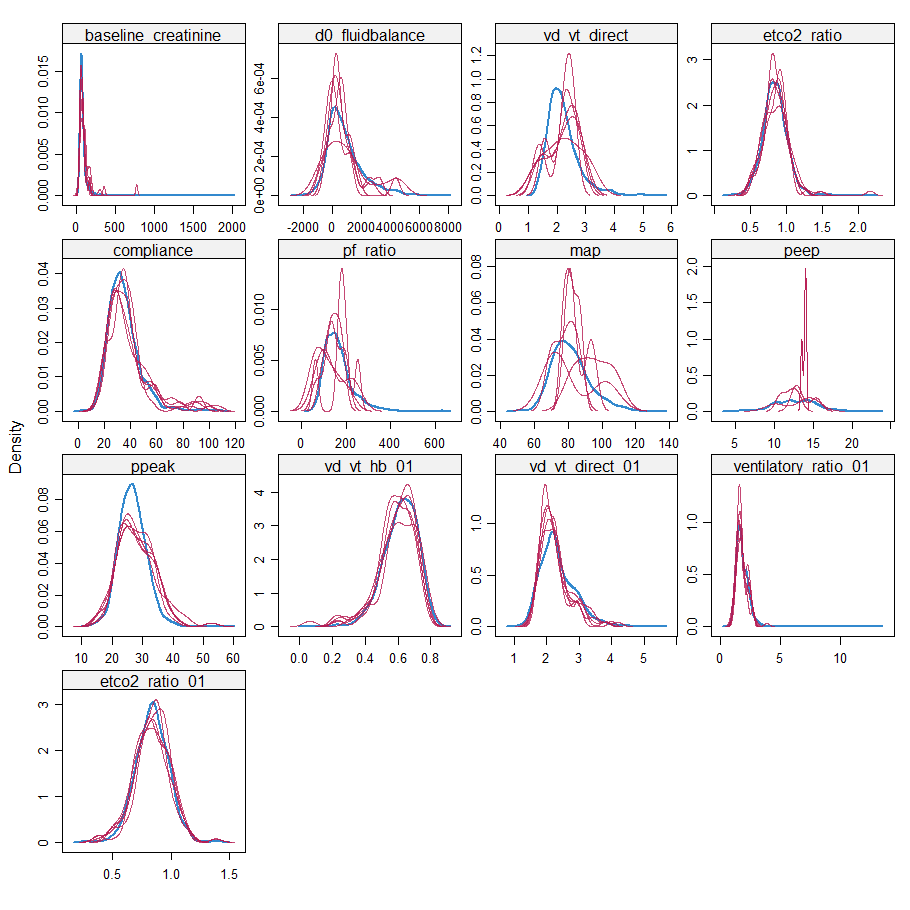
**

Blue line is non-imputed and red lines imputed values

**
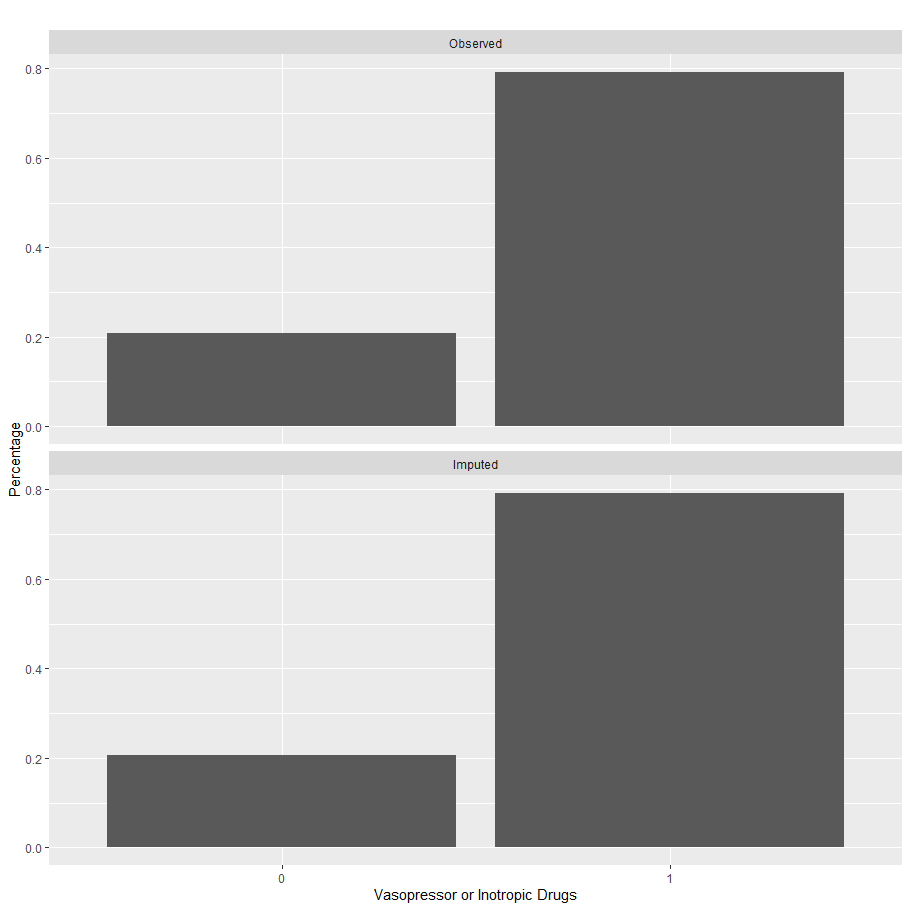
eFigure 3 - Categorical Variables Before and After Multiple Imputation**

**eFigure 4. Corrected Minute Ventilation in survivors vs non-survivors (left figure) and categorized by tertiles (right figure)**


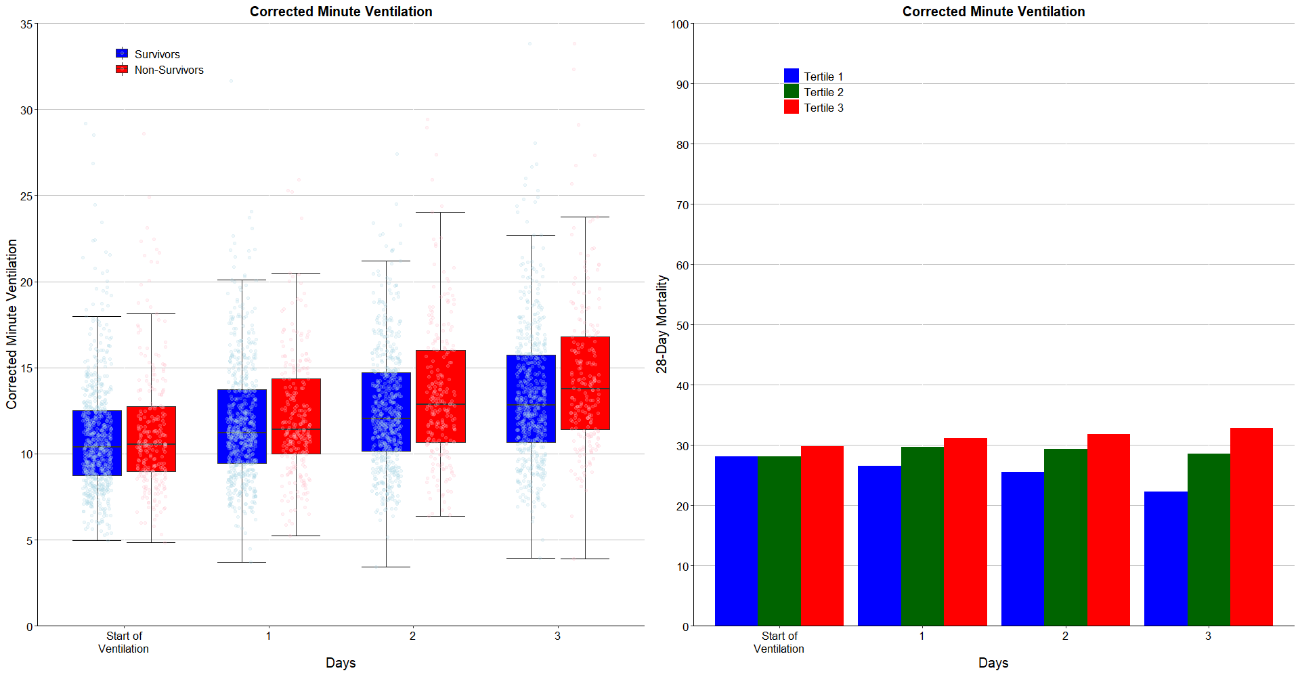


**eFigure 5. Kaplan–Meier Plot of the Cumulative Incidence of Corrected Minute Ventilation from start of ventilation up to day 28**


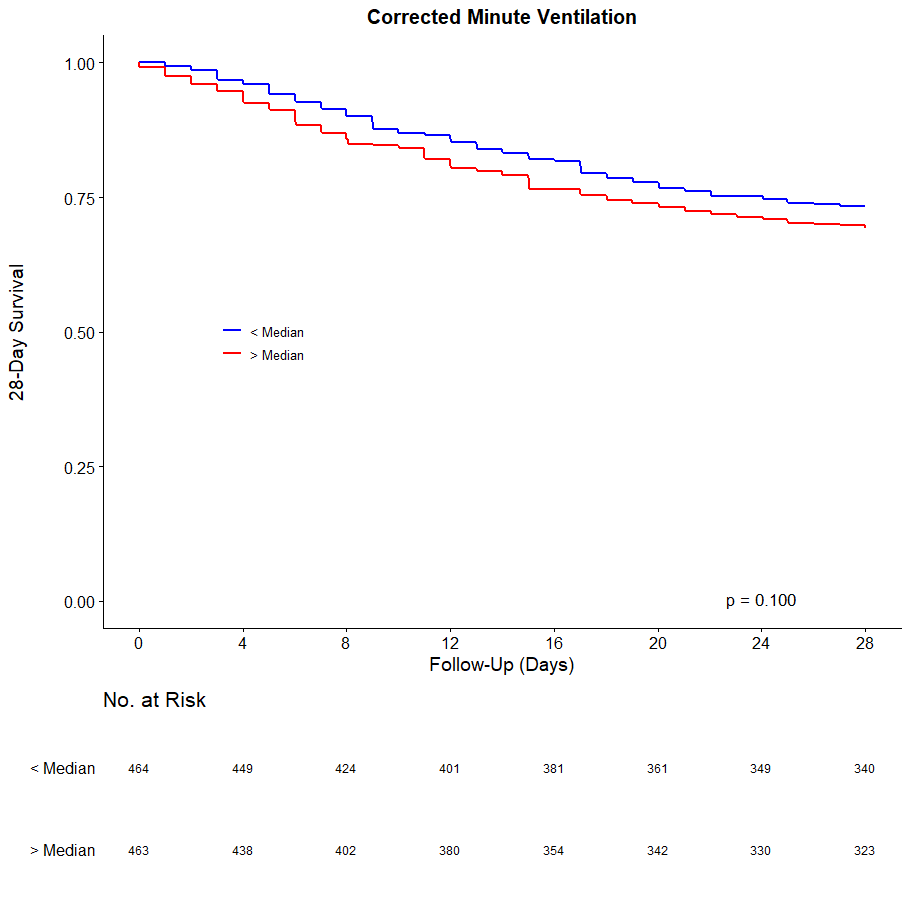

Supplement: Supplementary file 1 — Additional file 1. Impaired ventilation is not associated with 28-day mortality in COVID-19 ARDS [file 13054_2021_3570_MOESM1_ESM.docx]
